# State E-Cigarette Flavor Restrictions and Tobacco Product Use in Youths and Adults

**DOI:** 10.1001/jamanetworkopen.2025.24184

**Published:** 2025-07-30

**Authors:** David Cheng, Boram Lee, Abra M. Jeffers, Maeve Stover, Lindsay Kephart, Ginny Chadwick, Gina R. Kruse, A. Eden Evins, Nancy A. Rigotti, Douglas E. Levy

**Affiliations:** 1 Biostatistics Center, Massachusetts General Hospital, Boston; 2 Department of Medicine, Harvard Medical School, Boston, Massachusetts; 3 Ewha Womans University, Seoul, Republic of Korea; 4 Mongan Institute Health Policy Research Center, Massachusetts General Hospital, Boston; 5 Tobacco Research and Treatment Center, Division of General Internal Medicine, Massachusetts General Hospital, Boston; 6 Division of General Academic Pediatrics, Massachusetts General Hospital for Children, Boston; 7 Heller School of Social Policy, Brandeis University, Waltham, Massachusetts; 8 Division of General Internal Medicine, University of Colorado School of Medicine, Aurora; 9 Center for Addiction Medicine, Department of Psychiatry, Massachusetts General Hospital, Boston; 10 Department of Psychiatry, Harvard Medical School, Boston, Massachusetts

## Abstract

**Importance:**

Seven states have policies restricting the sale of flavored e-cigarettes. Limited evidence exists regarding these policies’ association with the use of tobacco products across age groups.

**Objective:**

To evaluate associations of e-cigarette flavor restriction policies with e-cigarette and cigarette use by age group over multiple years.

**Design, Setting, and Participants:**

In this cross-sectional study, annual state-level prevalences of e-cigarette and cigarette use in the US between 2015 and 2023 were separately estimated among youths (high school age) using data from the Youth Risk Behavior Survey (YRBS) and young adults (ages 18-24 years) and adults (ages ≥25 years) using the Behavioral Risk Factor Surveillance System (BRFSS). Six policy states had multiple years of postpolicy data available; other states were control states. Associations between policy and prevalence of e-cigarette and cigarette use in each postpolicy year were estimated annually using difference-in-differences analysis, setting 2019 as the prepolicy year and 2020 to 2023 as postpolicy years.

**Exposure:**

Statewide policy restricting sales of non–tobacco flavored e-cigarettes.

**Main Outcomes and Measures:**

State annual prevalence of past 30-day (YRBS) and current (BRFSS) e-cigarette and cigarette use.

**Results:**

Prevalence data were obtained for 186 (YRBS) and 386 (BRFSS) state-years for e-cigarettes and 191 (YRBS) and 456 (BRFSS) state-years for cigarettes. In recent years, e-cigarette use prevalence decreased among youths (eg, the change in mean prevalence from 2019 to 2023 was 24.1% to 14.0% for policy states and 24.6% to 17.2% for control states) but increased in control states among young adults (eg, mean prevalence, 17.0% in 2019 to 20.4% in 2023). Cigarette use prevalence decreased in policy and control states, although policy states exhibited lower prepolicy prevalence and attenuated postpolicy decreases (eg, mean prevalence, 6.7% in 2019 to 3.8% in 2023 among young adults) relative to control states (eg, mean prevalence, 12.1% in 2019 to 6.3% in 2023 among young adults). Flavor policies were associated with reduced e-cigarette use among young adults in 2022 (average treatment effect among the treated [ATT], −6.7 percentage points; 95% CI, −1.3 to −12.1 percentage points) and adults aged 25 years or older in 2023 (ATT, −1.2 percentage points; 95% CI, −2.0 to −0.4 percentage points) and increased cigarette use among youths in 2021 (ATT, 1.8 percentage points; 95% CI, 0.7 to 2.9 percentage points) and young adults in 2021 (ATT, 3.7 percentage points; 95% CI, 2.2 to 5.2 percentage points), 2022 (ATT, 2.7 percentage points; 95% CI, 1.4 to 4.1 percentage points), and 2023 (ATT, 3.2 percentage points; 95% CI, 0.9 to 5.5 percentage points).

**Conclusions and relevance:**

In this study, flavor restriction policies were associated with some reductions in e-cigarette use but also unintended increases in cigarette use, highlighting a need for further work evaluating potential substitution outcomes and prevention of tobacco use among youths.

## Key Points

**Question:**

Are statewide e-cigarette flavor restriction policies associated with e-cigarette and cigarette use among different age groups?

**Findings:**

In this cross-sectional study of 2019-2023 survey data on US high school-aged youths, young adults (ages 18-24 years), and adults ages 25 years or older, flavor policies were associated with reduced e-cigarette use among young adults and adults aged 25 years or older and increased cigarette use among youths and young adults, although results varied by state and year.

**Meaning:**

This study found that flavor policies were associated with reductions in e-cigarette use and unintended increases in cigarette use, highlighting a need for future work on evaluating substitution effects and prevention of youth tobacco use.

## Teaser

This survey study investigates the association of state e-cigarette flavor restriction policies with e-cigarette and cigarette use among youths, young adults, and adults.

## Introduction

The popularity of e-cigarettes among nonsmoking youths and young adults presents a significant public health concern. Recent estimates indicate that 1.2 million high school students (7.8%) and 11.1 million adults aged 18 years or older (4.5%) use e-cigarettes.^1,2,3^ Most e-cigarettes contain nicotine, an addictive substance associated with cognitive and behavioral impairments during adolescent brain development.^4^ E-cigarette use has also been associated with increased pulmonary and cardiovascular risks and mental health issues.^5^ Youth use of e-cigarettes may lead to symptoms of nicotine dependence and subsequent cigarette use.^6,7,8^ However, individuals smoking combustible cigarettes may benefit from switching to e-cigarettes as a harm reduction or smoking cessation tool.^9^

E-cigarettes with nontobacco flavors, such as fruit, candy, or alcohol (henceforth, *flavored* e-cigarettes), are marketed to appeal to youths and young adults.^4,10^ Among middle and high school students who used e-cigarettes, 87.6% used flavored products.^1^ To curb youth use, federal, state, and local authorities have enacted policies restricting the sale of flavored e-cigarettes. In early 2020, the US Food and Drug Administration imposed regulations against cartridge-based e-cigarettes with nontobacco and nonmenthol flavors. Adolescents quickly switched to disposable products, which were exempted.^11^ Currently, 7 states (Massachusetts, New Jersey, New York, Rhode Island, Utah, Maryland, and California) plus Washington, District of Columbia (DC) and more than 390 localities have enacted flavor restriction policies.^12^ These policies vary in scope and implementation, with some policies exempting mint and menthol products, specialty stores, or online sales.

Evidence for the association of flavor policies with e-cigarette and cigarette use is still emerging. Studies of local restrictions in Minnesota and Massachusetts found associations with reduced use of e-cigarettes and cigarettes.^13,14^ An early evaluation of statewide flavor policies in 4 states found associations with reduced short-term sales of e-cigarettes.^15^ Flavor policies are associated with unintended outcomes, such as encouraging substitution toward combustible cigarettes.^16,17^ Additional evaluations of specific state policies corroborated associations with reduced e-cigarette sales,^18,19,20^ although short-term increases in sales of tobacco-flavored e-cigarettes and cigarettes were observed.^19^ In a study of adults aged 18 to 59 years, restrictions were associated with reduced use of e-cigarettes and cigarettes.^21^ Among young adults aged 18 to 29 years, flavor policies have been associated with reduced use of e-cigarettes but increased use of cigarettes.^22^ Two 2025 preprint study publications^23,24^ also evaluated statewide policies across age groups using Youth Risk Behavior Survey (YRBS) and Behavioral Risk Factor Surveillance System (BRFSS) data, finding similar results. However, these studies did not include data from the latest prepolicy year, 2019, and did not consider underlying use trends.

This study sought to evaluate associations between flavor restriction policies and the prevalence of e-cigarette and cigarette use across age groups. We also assessed trends in e-cigarette and cigarette use over time and considered whether associations between policy and tobacco use were heterogeneous across states. To assess the evidence for substitution toward combustible cigarettes, we evaluated associations with the prevalence of cigarette use among individuals currently, formerly, and never using e-cigarettes. These data may contribute evidence on the association of flavor policies with e-cigarette and cigarette use and help inform policy decisions in other states.

## Methods

This cross-sectional study was deemed exempt from review and consent by the Mass General Brigham Institutional Review Board because it used publicly available secondary data. The study adhered to the Strengthening the Reporting of Observational Studies in Epidemiology (STROBE) reporting guideline.

### Data Sources and Study Populations

The YRBS and BRFSS are school- and telephone-based cross-sectional surveys, respectively, administered by states with support from the Centers for Disease Control and Prevention (CDC) to monitor health behaviors among US adolescents and adults.^25^ We used state-level data from both surveys from 2015 to 2023, including biennial data for high school students in grades 9 to 12 (YRBS) and annual data for adults^3^ aged 18 years or older (BRFSS). See the eAppendix in Supplement 1 for information on required disclosures regarding YRBS and BRFSS files.

### Current E-Cigarette and Cigarette Use

Questions on cigarette use were consistently asked in almost all states and years in both surveys (eTables 1 and 2 in Supplement 1). Questions on e-cigarette use were asked in nearly all states and survey years in YRBS and in 2016 to 2017 and 2021 to 2023 as part of the core BRFSS questionnaire. In 2015 and 2018 to 2020, questions on e-cigarette use were asked for select states as part of state-added or optional BRFSS modules. We refer to YRBS respondents as *youths* and BRFSS respondents aged 18 to 24 years and 25 years or older as *young adults* and *adults*, respectively.

Among YRBS respondents, current use of e-cigarettes or cigarettes was defined as use in at least 1 of the past 30 days. Among BRFSS respondents, current use of e-cigarettes or cigarettes was defined as use on “some days” or “every day.” For each state, year, and age group, prevalence was calculated using survey weights to obtain state-representative estimates. Current cigarette use prevalence among individuals currently, formerly, and never using e-cigarettes was also calculated in each state and year by age group. Former e-cigarette use was defined as ever but not current use of e-cigarettes. Respondents with missing, refused, or “don’t know” responses were excluded.

### Respondent Characteristics

For descriptive purposes, state-level data on respondent characteristics were summarized from 2019 and 2021 surveys. Characteristics included age, sex, school grade (9-12 for the youth population), self-reported race and ethnicity (Black or African American; Hispanic, Latino, or Spanish origin; White; multiracial (ie, chose >1 category); or additional race or ethnicity categories), current alcohol use, and current cannabis use. Additional race categories included American Indian or Alaska Native, Asian, Pacific Islander, or other, which were combined as *additional race or ethnicity categories* because of small population numbers.

### Statewide E-Cigarette Flavor Restriction Policies

We identified 6 policy states with permanent restrictions prohibiting the sale of flavored e-cigarettes: Massachusetts (in effect December 2019), New Jersey (April 2020), New York (May 2020), Maryland (February 2020), Utah (July 2020), and Rhode Island (March 2020).^12,26^ Washington, DC, and California enacted policies in October and December 2022, respectively, but were not included among policy states given that they did not have sufficient postpolicy data available to enable evaluations over multiple years. They were included as control states through 2022. Flavor policies varied in scope and implementation (Table 1).^27,28,29,30^ In particular, Maryland and Utah exempted menthol-flavored products. Utah additionally exempted mint-flavored products and sales in tobacco specialty stores. States without statewide flavor restrictions were considered control states.

**Table 1.** State-Level Flavor Restriction and Other E-Cigarette Policies, 2019-2023^a^

State	Flavor policy effective date	Legislation or administrative rule	E-cigarette flavor exemptions	Other flavor policy exemptions	Covers all tobacco products?^b^	State law prohibits online e-cigarette sales	T21 effective date	E-cigarette tax rate and effective date	Licensure required for e-cigarette retailers effective date	E-cigarette clean indoor air laws effective date^c^
MA	Sep 24, 2019 (emergency rule); Dec 11. 2019 (legislation)	Legislation	None	Exempts adult-only smoking bars for onsite consumption	Yes, restricts all flavored tobacco products (including menthol cigarettes, cigars, and others)	Prohibits shipping e-cigarettes without age verification or signature and online sales of flavored e-cigarettes	Dec 31, 2018	June 1, 2020; 75% wholesale	June 1, 2020	Dec 31, 2018
UT	Jul 1, 2020	Legislation	Mint and menthol	Exempts adult-only specialty tobacco stores	No, permits sales of other flavored tobacco products.	Yes, e-cigarette sales to UT consumers must be made face to face	Jul 1, 2020	July 1, 2020; 56% manufacturer’s sales price	July 1, 2015	May 8, 2012
NJ	April 20, 2020	Legislation	None	None	No, permits sales of other flavored tobacco products	None	Nov 1, 2017	Sep 30, 2018; $0.10/fluid mL; container e-liquid taxed at 10% retail price	Jun 30, 2019	Jul 11, 2010
NY	May 18, 2020	Legislation	None	Exempts products with a premarket FDA authorization	No, permits sales of other flavored tobacco products	Yes, prohibits shipping products to a consumer within the state	Nov 13, 2019	Dec 1, 2019; 20% retail price	Dec 1, 2019	Nov 22, 2017
RI	Oct 4, 2019 (emergency rule); Mar 26, 2020 (final rule)	Administrative rule (turned case law)	None	None	No, permits sales of other flavored tobacco products	Prohibits shipping e-cigarettes without age verification and signature	July 7, 2021	NA	Jan 1, 2015	Jul 1, 2018
MD	Feb 10, 2020	Administrative rule	Menthol	Rule targets cartridge-based and disposable e-cigarettes	No, permits sales of other flavored tobacco products	None	Oct 1, 2019	Mar 14, 2021; devices: 12% taxable price; vaping liquid ≤5 mL: 60% taxable price	Oct 1, 2017	NA

Abbreviations: FDA, Food and Drug Administration; T21, tobacco 21 laws restricting e-cigarette or cigarette sales to individuals younger than 21 years; NA, not applicable.

^a^ Policy details were obtained from the Public Health Law Center^27,28^ and Centers for Disease Control and Prevention.^29^

^b^ The 2009 Family Smoking Prevention and Tobacco Control Act banned flavored cigarettes but exempted menthol cigarettes. Sales of flavored cigarettes (apart from menthol) are not permitted in any state per federal law.^30^

^c^ Clean indoor air laws prohibiting e-cigarette use at restaurants, workplaces, and bars.

### Other Tobacco Control Policies

For sensitivity analyses, we also identified years in which specific states implemented other tobacco control policies, including e-cigarette taxes, a minimum legal sales age of 21 years for e-cigarettes, licensure requirement for e-cigarette sales, and prohibition of e-cigarette use in workplaces, restaurants, and bars (clean indoor air laws) (Table 1). Data on tobacco control policies and their effective dates were obtained from the CDC State Tobacco Activities Tracking and Evaluation (STATE) system,^31^ Public Health Law Center,^26^ and Campaign for Tobacco-Free Kids.^12^

### Statistical Analysis

Trends in mean state-level prevalence of e-cigarette and cigarette use were plotted from 2015 to 2023 using locally estimated scatterplot smoothing,^32^ separately for policy and control states by age group, to establish context. In primary analyses, we used difference-in-differences analysis to evaluate associations between flavor policies and the prevalence of current e-cigarette and cigarette use using state-level panel data from 2019 to 2023. Specifically, we fit linear regression models:

*Y_i_*_t_ = β_0_ + β_1_*X_i_* + β_2_*Z_t_* + β_3_*X_i_Z_t_* + ε_it_,

where *Y_it_* is the prevalence in state *i* in period *t* = 0 (prepolicy year, 2019) or period *t* = 1 (individual postpolicy years from 2020-2023), *X_i_* is an indicator of a policy (*X_i_* = 1) or control (*X_i_* = 0) state, *Z_t_* is an indicator of whether period *t* is a postpolicy period, and ε_it_ is a mean-zero error term. In this model, β_3_ is the parameter of interest, denoting the difference of the mean change in the prevalence from 2019 to the specified postpolicy year between policy and control states. Estimates of β_3_ can also be interpreted as the difference between observed and expected prevalence among policy states if trends relative to control states had been maintained in the absence of the policy (ie, the average treatment effect among the treated [ATT] under the parallel trends assumption).^33^ While the exact parallel trends assumptions cannot be verified empirically,^34^ the plausibility of parallel trends was evaluated through visual inspection of prepolicy trends and event-study plots. Given that policies were considered to be enacted in a single year (2020), methods for estimating outcomes associated with staggered policies were not needed.^35,36^ Separate models were fit for different age groups and postpolicy years 2020 to 2023. We allowed for unbalanced data, incorporating all state-years where outcome data were available. Robust standard errors accounting for state-level clustering with small-sample corrections were used to construct CIs and *P* values.^37^ Hypothesis tests against the null hypothesis that ATT estimates were zero were conducted using 2-sided *P* values at the α = .05 significance level.

In secondary analyses, we estimated difference-in-differences models excluding Maryland and Utah (due to their mint and menthol exemptions) and separately estimated state-specific associations by including data only from individual policy states and comparing with control states. We also repeated primary analyses focusing on prevalence of current cigarette use among respondents with current, former, and never use of e-cigarettes. We conducted sensitivity analyses restricting the sample to a balanced panel of states for each corresponding postpolicy year, using 2017 instead of 2019 as the prepolicy year (data on e-cigarette use were more complete in 2017), and including only states in years when other specific tobacco control policies were present or absent. Data were prepared and analyzed using R statistical software version 4.4.0 (R Project for Statistical Computing).

## Results

### Descriptives and Trends Analyses

We obtained data for 186 (YRBS) and 386 (BRFSS) state-years on e-cigarette use and 191 (YRBS) and 456 (BRFSS) state-years on cigarette use (eTables 1 and 2 in Supplement 1). Sample sizes and demographics varied across age groups, states, and years (eTable 3 in Supplement 1). For example, 6 policy states and 37 control states contributed data on e-cigarette use from 161 835 youths in 2019, while 5 policy states (New Jersey lacked 2019 data) and 23 control states contributed data from 10 882 young adults and 185 080 adults for that year. In 2019, the mean state-level age was 16.0 years for policy and control states among youths, 21.0 years for policy and 20.9 years for control states among young adults, and 51.1 years for policy and 51.7 years for control states among adults. The mean proportion of male respondents in 2019 was 50.8% for policy and 51.2% for control states among youths, 50.6% for policy and 51.9% for control states among young adults, and 48.0% for policy and 48.6% for control states among adults. Mean proportions in 2019 of Black only, Hispanic only, or White only respondents were 13.7%, 21.0%, and 55.3%, respectively, in policy states and 13.0%, 16.9%, and 58.4%, respectively, in control states for youths; 11.9%, 16.3%, and 58.5%, respectively, in policy states and 10.7%, 16.4%, and 61.1%, respectively, in control states for young adults; and 11.1%, 13.2%, and 67.7%, respectively, for policy states and 10.5%, 9.8%, and 72.0%, respectively, in control states for adults. Missingness in current e-cigarette and cigarette use did not differ appreciably for policy vs control states (eTable 4 in Supplement 1).

**Figure 1.**
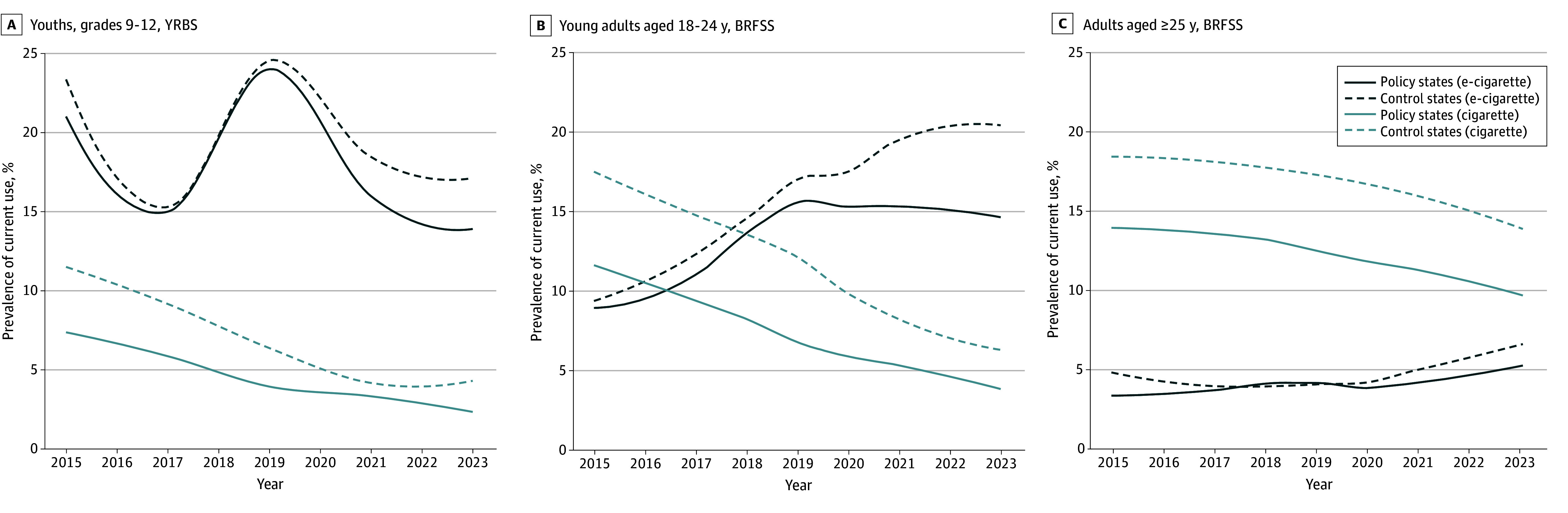
Smoothed Trends in Current E-Cigarette and Cigarette Use Current e-cigarette and cigarette use prevalence was estimated based on self-reported past 30-day use in youths and use on some days or every day in young adults and adults aged 25 years or older. Respondents with missing, refused, or “don’t know” responses were excluded. Prevalence was calculated using complex survey weights to obtain state-representative estimates in each year. Smoothed trends were estimated by locally estimated scatterplot smoothing with state-level biennial (Youth Risk Behavior Survey [YRBS]) and annual (Behavioral Risk Factor Surveillance System [BRFSS]) prevalence of e-cigarette and cigarette use among policy and control states by age group.

The prevalence of e-cigarette and cigarette use generally trended in the same direction for policy and control states, with differential rates of change (Figure 1; eFigure 1 in Supplement 1). Among youths, the mean state prevalence of current e-cigarette use peaked in 2019, then decreased (eg, 24.1% in 2019 to 14.0% in 2023 for policy states and 24.6% in 2019 to 17.2% in 2023 for control states). Among young adults, recent mean e-cigarette use prevalence increased in control states (eg, 17.0% in 2019 to 20.4% in 2023) but stabilized in policy states (eg, 15.6% in 2019 to 14.6% in 2023). Among adults, mean e-cigarette use prevalence was at lower levels compared with that among youths and young adults but increasing in recent years, more slowly in policy states (eg, 4.1% in 2019 to 5.2% in 2023 for policy states and 4.0% in 2019 to 6.6% in 2023 for control states). The mean prevalence of cigarette use was higher among adults than other age groups but essentially decreased over 2015 to 2023 in all populations (eg, 13.9% in 2015 to 9.7% in 2023 for policy states and 18.5% in 2015 to 13.9% in 2023 for control states). For youths and young adults, recent decreases in mean cigarette use were attenuated among policy states (eg, 6.7% in 2019 to 3.8% in 2023 for young adults) compared with control states (eg, 12.1% in 2019 to 6.3% in 2023 for young adults).

### Primary Difference-in-Differences Analyses

There were no clear departures from parallel trends based on prepolicy e-cigarette and cigarette use prevalence (Figure 1). Event-study plots confirmed that prepolicy differences in prevalence between policy and control states from 2015 to 2018 did not differ significantly from those in 2019 (eFigure 2 in Supplement 1).

Difference-in-differences estimates indicated that flavor policy was associated with a reduction below expected trends in e-cigarette prevalence among young adults in 2022 (ATT, −6.7 percentage points; 95% CI, −12.1 to −1.3 percentage points) and adults in 2023 (ATT, −1.2 percentage points; 95% CI, −2.0 to −0.4 percentage points) (Table 2). While cigarette use prevalence generally decreased in policy and control states, the policy was associated with increases above expected trends among youths in 2021 (ATT, 1.8 percentage points; 95% CI, 0.7 to 2.9 percentage points) but not in 2023 and in young adults in 2021 (ATT, 3.7 percentage points; 95% CI, 2.2 to 5.2 percentage points), 2022 (ATT, 2.7 percentage points; 95% CI, 1.4 to 4.1 percentage points), and 2023 (ATT, 3.2 percentage points; 95% CI, 0.9 to 5.5 percentage points). There were no associations with cigarette use prevalence among adults.

**Table 2.** Association of Flavor Restriction Policy With Current E-Cigarette and Cigarette Use

Year	Current e-cigarette use^a^^,^^b^		Current cigarette use^a^^,^^b^	
	ATT (95% CI)	*P* value	ATT (95% CI)	*P* value
**Youths, grades 9-12, YRBS**				
2021	−0.019 (−0.074 to 0.035)	.43	0.018 (0.007 to 0.029)	.007
2023	−0.027 (−0.070 to 0.017)	.19	0.006 (−0.004 to 0.017)	.19
**Young adults aged 18-24 y, BRFSS**				
2020	−0.041 (−0.093 to 0.012)	.11	0.014 (−0.001 to 0.028)	.06
2021	−0.044 (−0.111 to 0.023)	.16	0.037 (0.022 to 0.052)	.001
2022	−0.067 (−0.121 to −0.013)	.02	0.027 (0.014 to 0.041)	.003
2023	−0.060 (−0.124 to 0.003)	.06	0.032 (0.009 to 0.055)	.01
**Adults aged ≥25 y, BRFSS**				
2020	−0.001 (−0.012 to 0.009)	.81	0.000 (−0.012 to 0.013)	.96
2021	−0.009 (−0.02 to 0.003)	.11	0.002 (−0.010 to 0.014)	.65
2022	−0.01 (−0.023 to 0.003)	.12	0.006 (−0.005 to 0.018)	.22
2023	−0.012 (−0.020 to −0.004)	.009	0.006 (−0.008 to 0.021)	.34

Abbreviations: ATT, average treatment effect among treated; BRFSS, Behavioral Risk Factor Surveillance System; YRBS, Youth Risk Behavior Survey.

^a^ Estimates are based on difference-in-differences analyses between policy (Massachusetts, New Jersey, New York, Rhode Island, Maryland, and Utah) and control states and prevalence of use in prepolicy (2019) and specified postpolicy years.

^b^ CIs and *P* values are based on cluster-robust standard errors clustered by state with small-sample correction.^37^

### State-Specific Analyses

**Figure 2.**
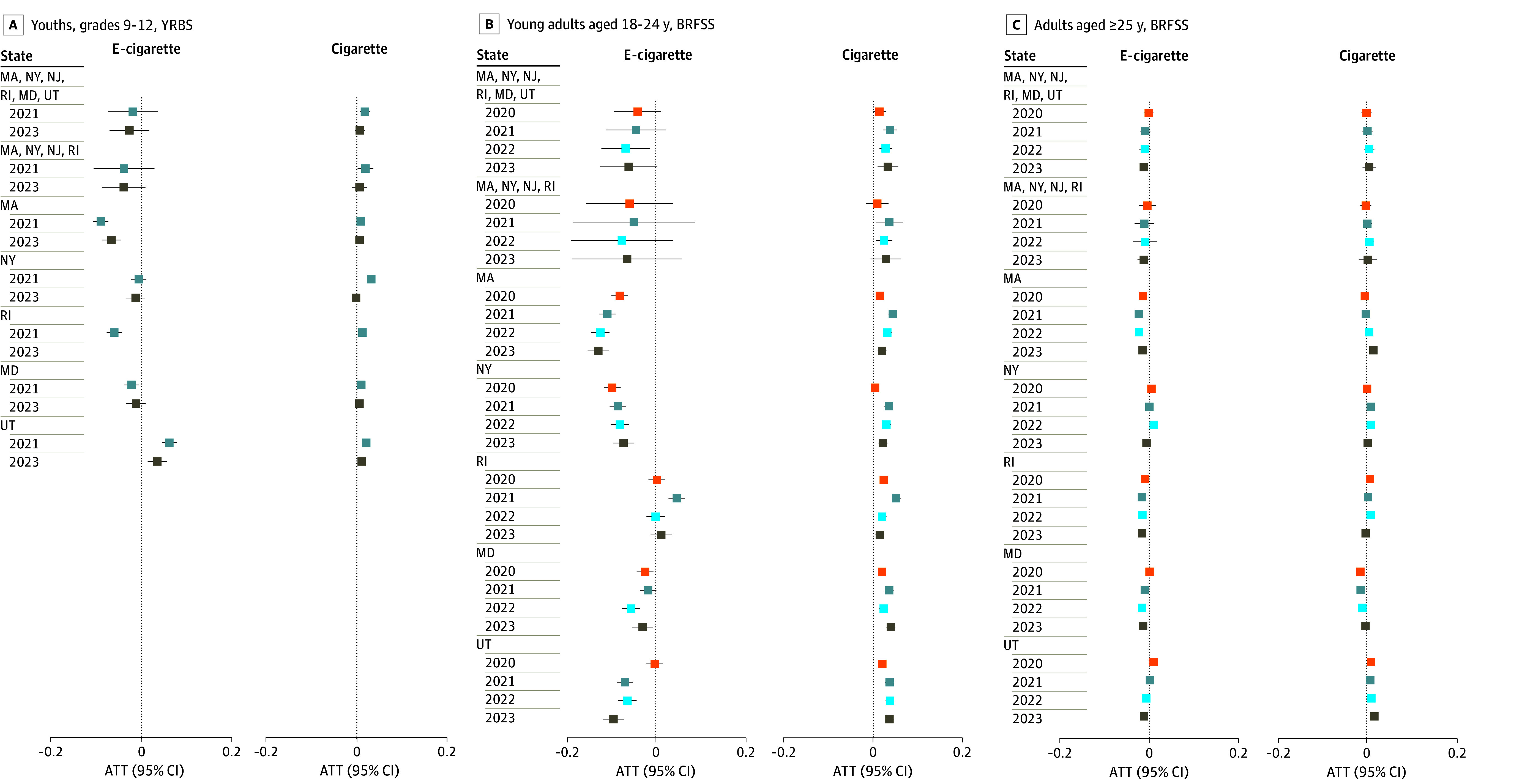
Association of Flavor Restriction Policy With Current E-Cigarette and Cigarette Use ATT indicates average treatment effect among the treated; BRFSS, Behavioral Risk Factor Surveillance System; YRBS, Youth Risk Behavior Survey.

There was heterogeneity in difference-in-differences estimates across states (Figure 2; eTable 5 in Supplement 1). The policy was associated with consistent reductions in e-cigarette use in Massachusetts and Maryland across postpolicy years and age groups; ATTs ranged from −12.8 percentage points (95% CI, −15.2 to −10.4 percentage points) in 2023 among young adults to −1.4 percentage points (95% CI, −1.8 to −1.1 percentage points) in 2020 among adults for Massachusetts and −5.5 percentage points (95% CI, −7.5 to −3.5 percentage points) in 2022 among young adults to 0.1 percentage points (95% CI, −0.3 to 0.5 percentage points) in 2020 among adults for Maryland. Associations with policies in other states were mixed. For example, there were significant reductions in e-cigarette use in Rhode Island among youths and adults; ATTs across postpolicy years and age groups ranged from −6.0 percentage points (95% CI, −7.7 to −4.4 percentage points) in 2021 among youths to −0.9 percentage points (95% CI, −1.3 to −0.6 percentage points) in 2020 among adults. However, there were either no significant reductions among young adults or, as in the case of 2021, a significant increase of 4.7 percentage points (95% CI, 2.8 to 6.5 percentage points). Policies were consistently associated with increased cigarette use across all policy states among youths and young adults, with some attenuation over time.

### Analyses of Cigarette Use by E-Cigarette Use

The policy was associated with increases in the prevalence of current cigarette use among individuals with current and former e-cigarette use in youths (eg, ATT among current e-cigarette users in 2021, 8.0 percentage points; 95% CI, 3.4-12.6 percentage points) and young adults (eg, ATT among former e-cigarette users in 2023, 8.3 percentage points; 95% CI, 1.6-15.0 percentage points). The policy was also associated with increased cigarette use among young adults who had never used e-cigarettes in 2023, although the point estimate was lower in magnitude (ATT, 2.2 percentage points; 95% CI, 0.3-4.0 percentage points); there were nonsignificant increases for young adults who had never used e-cigarettes in 2020-2022. There were nonsignificant increases for youths and adults who never used e-cigarettes in all years assessed (eTable 6 in Supplement 1).

### Sensitivity Analyses

**Figure 3.**
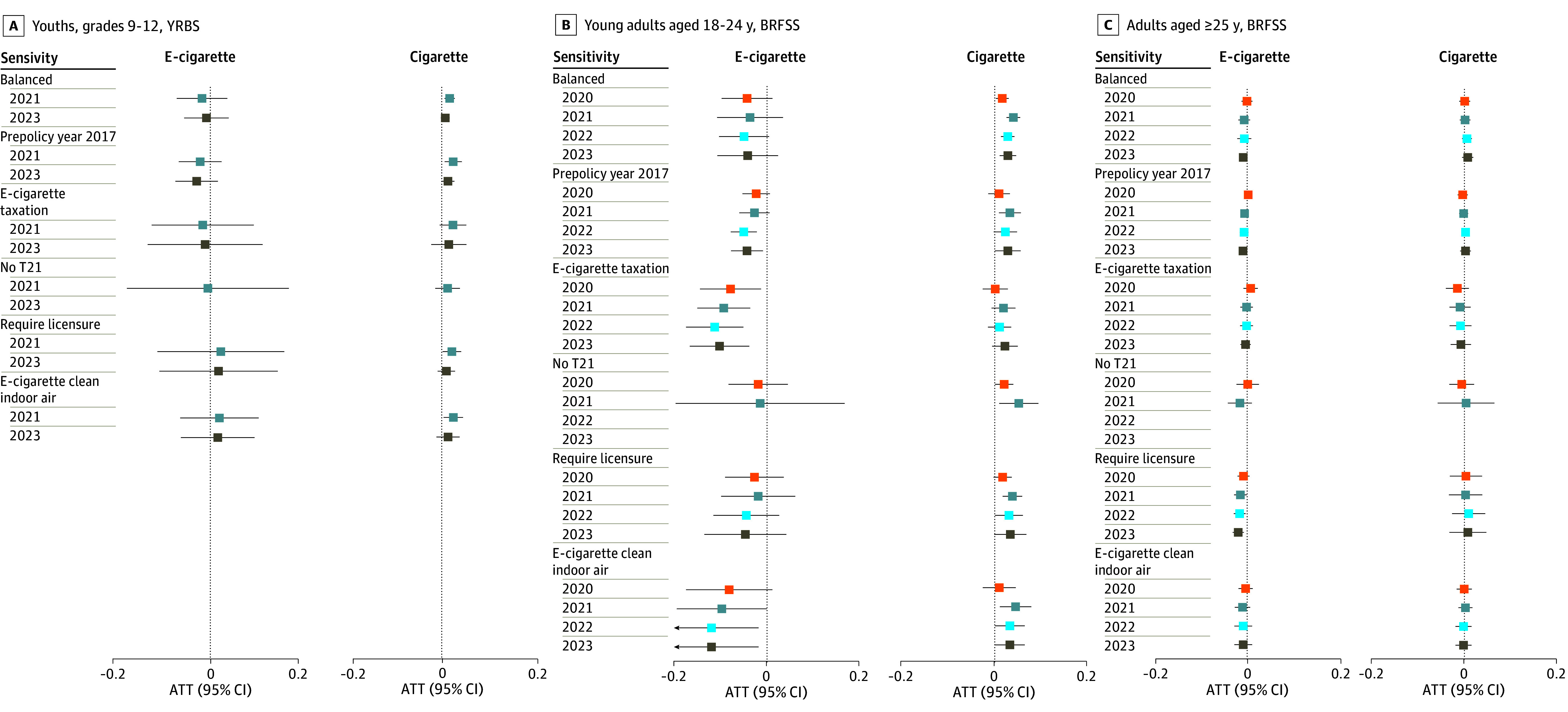
Sensitivity Analyses for Association of Flavor Restriction Policy With Current E-Cigarette and Cigarette Use Balanced analysis was restricted to states with a balanced panel for each postpolicy year. Prepolicy year 2017 analysis used 2017 instead of 2019 as the prepolicy year. E-cigarette taxation analysis was restricted to states in years with e-cigarette taxes present. No T21 (tobacco 21) analysis was restricted to states in years without tobacco 21 laws restricting sales of e-cigarettes and cigarettes for individuals aged 18 to 20 years. Require licensure analysis was restricted to states in years requiring licensure to sell e-cigarettes. E-cigarette clean indoor air analysis was restricted to states in years with prohibitions on e-cigarette use at restaurants, workplaces, and bars. ATT indicates average treatment effect among the treated; BRFSS, Behavioral Risk Factor Surveillance System; YRBS, Youth Risk Behavior Survey.

In sensitivity analyses (Figure 3; eTables 7 and 8 in Supplement 1), policy associations were generally consistent with those of primary analyses, exhibiting reduced e-cigarette use among young adults and adults and increased cigarette use among youths and young adults. A notable exception was the association of the policy with reduced e-cigarette use and the nonsignificant differences in cigarette use by policy status among young adults in states with e-cigarette taxation.

## Discussion

In difference-in-differences analyses in this cross-sectional study found that state flavor restriction policies were associated with reductions in e-cigarette use in 2022 among young adults and in 2023 among adults 25. These results are consistent with previous studies that found associations with reduced e-cigarette sales^15,18,19^ and use.^13,14,23,24,38,39^ While associations in 2020 were expected to be diminished given that the policy was not in place in most states for the full year, the lack of associations prior to 2022 suggests a potential delay in observable associations with behavior after policy enactment due to time required to implement policies and set up enforcement.

The prevalence of cigarette use decreased from 2015 to 2023, reaching less than 10% in recent years among youths and young adults. These decreases were attenuated among policy states after 2019. Accordingly, difference-in-differences estimates in primary analyses indicate that flavor restrictions were associated with increased cigarette use among youths and young adults, with the exception of youth use in 2023. Similar associations have been identified in recent studies,^17,22,23,24,40^ although the underlying reasons remain unclear. Given that prepolicy cigarette use prevalence was already low among policy states, the prevalence may be approaching a floor in which subsequent decreases are muted. However, young adults have reported in surveys that they would switch to cigarettes if access to flavored e-cigarettes became restricted.^16,41^

For youths, analysis of cigarette use prevalence among respondents by current, former, and never e-cigarette use revealed that policy-associated increases in cigarette use occurred primarily in individuals with current and former e-cigarette use. This suggests that associations with cigarette use may be contributed by increased dual use and substitution from e-cigarettes rather than uptake among individuals naïve to e-cigarettes. Among young adults, there were late, positive associations among respondents who never used e-cigarettes, although the magnitude of ATTs was much lower than among individuals who formerly used e-cigarettes. Further work using longitudinal data is needed to more directly assess rates of substitution from e-cigarettes to cigarettes in response to flavor restrictions. Considering these potential unintended outcomes associated with flavor policies, research is needed to develop effective vaping cessation tools to enable youths and young adults to successfully quit all tobacco products when the availability of flavored products becomes limited.^42^

Associations with e-cigarette use exhibited clear variation across states, populations, and time periods. In some cases, this variation can be explained by known contextual and implementation details. For example, Massachusetts exhibited early reductions in e-cigarette use associated with the policy and sustained reductions in subsequent years across all populations. This state had issued a temporary ban on all e-cigarette sales in September 2019, prior to the statewide flavor ban in December. Additionally, many local jurisdictions in Massachusetts had already initiated restrictions on flavored e-cigarettes,^43^ laying the groundwork for policy implementation and enforcement. Recent work has highlighted an association between the Maryland policy and reduced cigarette use among young adults aged 18 to 29 years.^22^ Although we observed associations of the policy with reduced cigarette use among adults aged 25 years or older, associations remained with increased cigarette use among youths and young adults aged 18 to 24 years. We are thus unable to conclude that any state policy completely avoided adverse outcomes.

### Limitations

This study has several limitations. Flavor policies adopted in 2020 coincided with the onset of the COVID-19 pandemic. Changes to e-cigarette and cigarette use due to the differential effects of the pandemic could also have induced some observed associations. YRBS data are typically collected in the spring, but in 2021, YRBS collection occurred in the fall due to the pandemic, creating misalignment in data seasonality across years. However, both policy and control states would be impacted by this shift. In our primary analyses, we set 2019 as the prepolicy year, although Rhode Island and Massachusetts had policies effective in October and December 2019, respectively. Nevertheless, results did not change when the prepolicy year was set to 2017.

These findings represent the experiences of the first several states to institute flavor restriction policies. Results may shift over time as new and updated policies adapt to the exigencies of current retail and regulatory environments. Although we focused on state-level policies in this study, local flavor policies may have an impact on states even if no state-level policy exists. There was a moderate degree of nonresponse or refusal to answer questions on current e-cigarette use or cigarette use, but there were no clear differences in these rates between policy and control states.

## Conclusions

In this cross-sectional study of statewide policies restricting flavored e-cigarettes, policies were associated with reduced e-cigarette use among young adults and adults aged 25 years or older but also with increased cigarette use among youths and young adults. Associations with cigarette use were largely attributable to increases among individuals currently and formerly using e-cigarettes. Estimates of policy associations varied across states, age groups, and years. Further investigation is needed to better understand the cause of increased cigarette use and identify implementation details that underlie successful policies.

## Notes

Supplement 1. **eAppendix.** Required disclosures for BRFSS and YRBS data **eTable 1.** Availability of e-cigarette and cigarette use data in Youth Risk Behavior Survey **eTable 2.** Availability of e-cigarette and cigarette use data in Behavioral Risk Factor Surveillance System **eTable 3.** Summary of state-level aggregate characteristics among policy and control states by age group **eTable 4.** Summary of survey nonresponse to e-cigarette and cigarette questions by age group **eTable 5.** Association of flavor restriction policy with current e-cigarette and cigarette use by age group and specific states (numerical estimates) **eTable 6.** Association of flavor restriction policy with current cigarette use by e-cigarette use history **eTable 7.** State-year inclusion of BRFSS and YRBS data in Figure 3 sensitivity analyses **eTable 8.** Sensitivity analyses for association of flavor restriction policy on current e-cigarette and cigarette use by age group (numerical estimates) **eFigure 1.** State-level trends in current e-cigarette and cigarette use over time by age group **eFigure 2.** Event-study plots for e-cigarette and cigarette ATT estimates by age group

Supplement 2.
Data Sharing Statement
