# Supplementary material for: State E-Cigarette Flavor Restrictions and Tobacco Product Use in Youths and Adults
Source: JAMA Netw Open. 2025 Jul 30;8(7):e2524184. doi: 10.1001/jamanetworkopen.2025.24184 (PMC12311715; doi:10.1001/jamanetworkopen.2025.24184)
Supplement: Supplement 1. — eAppendix. Required disclosures for BRFSS and YRBS data eTable 1. Availability of e-cigarette and cigarette use data in Youth Risk Behavior Survey eTable 2. Availability of e-cigarette and cigarette use data in Behavioral Risk Factor Surveillance System eTable 3. Summary of state-level aggregate characteristics among policy and control states by age group eTable 4. Summary of survey nonresponse to e-cigarette and cigarette questions by age group eTable 5. Association of flavor restriction policy with current e-cigarette and cigarette use by age group and specific states (numerical estimates) eTable 6. Association of flavor restriction policy with current cigarette use by e-cigarette use history eTable 7. State-year inclusion of BRFSS and YRBS data in Figure 3 sensitivity analyses eTable 8. Sensitivity analyses for association of flavor restriction policy on current e-cigarette and cigarette use by age group (numerical estimates) eFigure 1. State-level trends in current e-cigarette and cigarette use over time by age group eFigure 2. Event-study plots for e-cigarette and cigarette ATT estimates by age group [file jamanetwopen-e2524184-s001.pdf]

Supplemental Online Content

Cheng D, Lee B, Jeffers AM, et al. State e-cigarette flavor restrictions and tobacco product use in youths and adults. *JAMA Netw Open*. 2025;8(7):e2524184. doi:10.1001/jamanetworkopen.2025.24184

eAppendix. Required disclosures for BRFSS and YRBS data

eTable 1. Availability of e-cigarette and cigarette use data in Youth Risk Behavior Survey

eTable 2. Availability of e-cigarette and cigarette use data in Behavioral Risk Factor Surveillance System

eTable 3. Summary of state-level aggregate characteristics among policy and control states by age group

eTable 4. Summary of survey nonresponse to e-cigarette and cigarette questions by age group

eTable 5. Association of flavor restriction policy with current e-cigarette and cigarette use by age group and specific states (numerical estimates)

eTable 6. Association of flavor restriction policy with current cigarette use by e-cigarette use history

eTable 7. State-year inclusion of BRFSS and YRBS data in Figure 3 sensitivity analyses

eTable 8. Sensitivity analyses for association of flavor restriction policy on current e-cigarette and cigarette use by age group (numerical estimates)

eFigure 1. State-level trends in current e-cigarette and cigarette use over time by age group

eFigure 2. Event-study plots for e-cigarette and cigarette ATT estimates by age group

This supplemental material has been provided by the authors to give readers additional information about their work.

© 2025 Cheng D et al. *JAMA Network Open*.

eAppendix. Required Disclosures for BRFSS and YRBS Data

Except as noted below, data were sourced from the Centers for Disease Control and Prevention (CDC). Behavioral Risk Factor Surveillance System Survey Data. Atlanta, Georgia: U.S. Department of Health and Human Services, Centers for Disease Control and Prevention, [2015, 2016, 2017, 2018, 2019, 2020, 2021, 2022] and the Centers for Disease Control and Prevention. [2015, 2017, 2019, 2021] Youth Risk Behavior Survey Data.

The analyses, interpretations and conclusions presented in this publication are the sole responsibilities of the listed authors, not of the state or federal departments from which these data were obtained.

State-added BRFSS data were individually obtained from specific states. The following disclosures are required for publications using the data.

1. Data for this publication were obtained in part from the Alaska Department of Health, Division of Public Health, Section of Chronic Disease Prevention and Health Promotion consisting of the Alaska Behavioral Risk Factor Surveillance System (BRFSS) Program dataset for the years of 2015, 2018, and 2019.
2. Data for this publication were obtained in part from the Arkansas Behavioral Risk Factor Surveillance System conducted under the direction of the Arkansas Department of Health. Funding for the BRFSS was provided in part by the U.S. Centers for Disease Control and Prevention. The interpretation and conclusions of the data are the sole responsibility of the author and not that of the ADH or CDC.
3. Data for this publication were obtained in part from the California Behavioral Risk Factor Surveillance System Program
4. Data for this publication were obtained in part from the CT Department of Public Health and Centers for Disease Control and Prevention (CDC)
5. Data for this publication were obtained in part from the Delaware Health and Social Services, Division of Public Health
6. Data for this publication were obtained in part from the District of Columbia Department of Health, Center for Policy, Planning and Evaluation, Behavioral Risk Factor Surveillance System (DC BRFSS), [2019]
7. The Florida Behavioral Risk Factor Surveillance System data used in this report were collected by the Florida Department of Health (FDOH). The views expressed herein are solely those of the authors and do not necessarily reflect those of the FDOH.
8. Data for this publication were obtained in part from the Kansas Department of Health and Environment. These analyses, conclusions, interpretations and recommendations drawn from these data are solely those of our research team members and do not represent the views of the Kansas Department of Health and Environment.
9. Data for this publication were obtained in part from the Massachusetts Department of Elementary and Secondary Education. MYRBS Dataset [2015, 2017]
10. This publication utilizes data provided by the Maryland Department of Health, Maryland Behavioral Risk Factor Surveillance System Program; collected under guidance of the Centers for Disease Control and Prevention; and analyzed by research team members affiliated with Massachusetts General Hospital. Its contents are solely the responsibility of the authors and do not necessarily represent the official views of the Maryland Department of Health or the Centers for Disease Control and Prevention.

© 2025 Cheng D et al. *JAMA Network Open*.

11. Data for this publication were obtained in part from the Michigan Department of Health and Human Services, our article authors assume full responsibility for the analysis and interpretation of the data
12. Data for this publication were obtained in part from the Missouri Behavioral Risk Factor Surveillance System conducted under the direction of the Missouri Department of Health and Senior Services (MDHSS) in collaboration with the University of Missouri-Columbia. Funding for the BRFSS was provided in part by the U.S. Centers for Disease Control and Prevention. The interpretation and conclusions of the data are the sole responsibility of the author and not that of the MDHSS or CDC.
13. Montana Behavioral Risk Factor Surveillance System, [2015, 2019, 2020] Behavioral Risk Factor Surveillance System Office, Montana Department of Public Health and Human Services, and supported by the Centers for Disease Control and Prevention Cooperative Agreements: 1U58DP006044-01, NU58DP006044-05. The contents are the sole responsibility of the authors.
14. BRFSS data used in these analyses were obtained from the Ohio Department of Health (ODH), supported by the Centers for Disease Control and Prevention (CDC). Use of these data does not imply that ODH or CDC agrees or disagrees with the analyses, interpretations or conclusions in this publication
15. Rhode Island Behavioral Risk Factor Surveillance System, [2015, 2017, 2018, 2019, 2022] Center for Health Data and Analysis, Rhode Island Department of Health, and supported in part by the National Center for Chronic Disease Prevention and Health Promotion, Centers for Disease Control and Prevention Cooperative Agreement Number, U58 DP006067
16. Data for this publication were obtained in part from the Tennessee Department of Health, Division of Vital Records & Vital Statistics and Division of Population Health Assessment. Behavioral Risk Factor Surveillance System Dataset [2019]
17. Data for this publication were obtained in part from the Office of Research and Evaluation. Utah Behavioral Risk Factor Surveillance System Survey Data. Salt Lake City, UT: Utah Department of Health and Human Services, [2015, 2017, 2018, 2019, 2022]

© 2025 Cheng D et al. *JAMA Network Open*.

eTable 1: Availability of E-Cigarette (EC) and Cigarette Use Data in Youth Risk Behavior Survey[a]

State	Question	2015	2017	2019	2021	2023
Alabama	E-cigarette use	Available	Not available	Available	Available	Not available
	Cigarette use	Available	Not available	Available	Available	Not available
Alaska	E-cigarette use	Available	Available	Available	Not available	Available
	Cigarette use	Available	Available	Available	Not available	Available
Arizona	E-cigarette use	Available	Available	Available	Available	Not available
	Cigarette use	Available	Available	Available	Available	Not available
Arkansas	E-cigarette use	Available	Available	Available	Available	Available
	Cigarette use	Available	Available	Available	Available	Available
California	E-cigarette use	Available	Available	Available	Not available	Not available
	Cigarette use	Available	Available	Available	Not available	Not available
Colorado	E-cigarette use	Not available	Available	Available	Available	Not available
	Cigarette use	Not available	Available	Available	Available	Not available
Connecticut	E-cigarette use	Not available	Not available	Available	Available	Not available
	Cigarette use	Available	Available	Available	Available	Not available
Delaware	E-cigarette use	Available	Available	Not available	Available	Not available
	Cigarette use	Available	Available	Not available	Available	Not available
D.C.	E-cigarette use	Not available	Not available	Not available	Not available	Not available
	Cigarette use	Not available	Not available	Not available	Not available	Not available
Florida	E-cigarette use	Not available	Not available	Not available	Not available	Not available
	Cigarette use	Available	Available	Available	Available	Not available
Georgia	E-cigarette use	Not available	Not available	Available	Available	Not available
	Cigarette use	Not available	Not available	Available	Available	Not available
Hawaii	E-cigarette use	Available	Available	Available	Available	Available
	Cigarette use	Available	Available	Available	Available	Available
Idaho	E-cigarette use	Available	Available	Available	Available	Not available
	Cigarette use	Available	Available	Available	Available	Not available
Illinois	E-cigarette use	Available	Available	Available	Available	Available
	Cigarette use	Available	Available	Available	Available	Available

© 2025 Cheng D et al. *JAMA Network Open*.

State	Question	2015	2017	2019	2021	2023
Indiana	E-cigarette use	Available	Not available	Not available	Available	Available
	Cigarette use	Available	Not available	Not available	Available	Available
Iowa	E-cigarette use	Not available	Available	Available	Available	Not available
	Cigarette use	Not available	Available	Available	Available	Not available
Kansas	E-cigarette use	Not available	Available	Available	Available	Not available
	Cigarette use	Not available	Available	Available	Available	Not available
Kentucky	E-cigarette use	Available	Available	Available	Available	Available
	Cigarette use	Available	Available	Available	Available	Available
Louisiana	E-cigarette use	Not available	Available	Available	Available	Not available
	Cigarette use	Not available	Available	Available	Available	Not available
Maine	E-cigarette use	Available	Available	Available	Available	Not available
	Cigarette use	Available	Available	Available	Available	Not available
**Maryland****	E-cigarette use	Available	Available	Available	Available	Available
	Cigarette use	Available	Available	Available	Available	Available
**Massachusetts***	E-cigarette use	Available	Available	Available	Available	Available
	Cigarette use	Available	Available	Available	Available	Available
Michigan	E-cigarette use	Available	Available	Available	Available	Available
	Cigarette use	Available	Available	Available	Available	Available
Minnesota	E-cigarette use	Not available	Not available	Not available	Not available	Not available
	Cigarette use	Not available	Not available	Not available	Not available	Not available
Mississippi	E-cigarette use	Available	Not available	Available	Available	Available
	Cigarette use	Available	Not available	Available	Available	Available
Missouri	E-cigarette use	Available	Available	Available	Available	Available
	Cigarette use	Available	Available	Available	Available	Available
Montana	E-cigarette use	Available	Available	Available	Available	Available
	Cigarette use	Available	Available	Available	Available	Available
Nebraska	E-cigarette use	Available	Available	Available	Available	Available
	Cigarette use	Available	Available	Available	Available	Available
Nevada	E-cigarette use	Available	Available	Available	Available	Available
	Cigarette use	Available	Available	Available	Available	Available

© 2025 Cheng D et al. *JAMA Network Open*.

State	Question	2015	2017	2019	2021	2023
New Hampshire	E-cigarette use	Available	Available	Available	Available	Available
	Cigarette use	Available	Available	Available	Not available	Available
**New Jersey***	E-cigarette use	Not available	Not available	Available	Available	Available
	Cigarette use	Not available	Not available	Available	Available	Available
New Mexico	E-cigarette use	Available	Available	Available	Available	Available
	Cigarette use	Available	Available	Available	Available	Available
**New York***	E-cigarette use	Available	Available	Available	Available	Available
	Cigarette use	Available	Available	Available	Available	Available
North Carolina	E-cigarette use	Available	Available	Available	Available	Available
	Cigarette use	Available	Available	Available	Available	Available
North Dakota	E-cigarette use	Available	Available	Available	Available	Available
	Cigarette use	Available	Available	Available	Available	Available
Ohio	E-cigarette use	Not available	Not available	Available	Available	Not available
	Cigarette use	Not available	Not available	Available	Available	Not available
Oklahoma	E-cigarette use	Available	Available	Available	Available	Available
	Cigarette use	Available	Available	Available	Available	Available
Oregon	E-cigarette use	Not available	Not available	Not available	Not available	Not available
	Cigarette use	Not available	Not available	Not available	Not available	Not available
Pennsylvania	E-cigarette use	Available	Available	Available	Available	Available
	Cigarette use	Available	Available	Available	Available	Available
**Rhode Island***	E-cigarette use	Available	Available	Available	Available	Not available
	Cigarette use	Available	Available	Available	Available	Not available
South Carolina	E-cigarette use	Available	Available	Available	Available	Not available
	Cigarette use	Available	Available	Available	Available	Not available
South Dakota	E-cigarette use	Available	Not available	Available	Available	Not available
	Cigarette use	Available	Not available	Available	Available	Not available
Tennessee	E-cigarette use	Available	Available	Available	Available	Available
	Cigarette use	Available	Available	Available	Available	Available
Texas	E-cigarette use	Not available	Available	Available	Available	Available
	Cigarette use	Not available	Available	Available	Available	Available

© 2025 Cheng D et al. *JAMA Network Open*.

State	Question	2015	2017	2019	2021	2023
**Utah****	E-cigarette use	Not available	Available	Available	Available	Available
	Cigarette use	Not available	Available	Available	Available	Available
Vermont	E-cigarette use	Available	Available	Available	Available	Available
	Cigarette use	Available	Available	Available	Available	Available
Virginia	E-cigarette use	Available	Available	Available	Available	Available
	Cigarette use	Available	Available	Available	Available	Available
Washington	E-cigarette use	Not available	Not available	Not available	Not available	Not available
	Cigarette use	Not available	Not available	Not available	Not available	Not available
West Virginia	E-cigarette use	Available	Available	Available	Available	Not available
	Cigarette use	Available	Available	Available	Available	Not available
Wisconsin	E-cigarette use	Not available	Available	Available	Available	Available
	Cigarette use	Not available	Available	Available	Available	Available
Wyoming	E-cigarette use	Available	Not available	Not available	Not available	Not available
	Cigarette use	Available	Not available	Not available	Not available	Not available
No. of available data state-years	E-cigarette use	35	37	43	43	28
	Cigarette use	37	39	44	43	28
No. of states contributing to policy group	E-cigarette use	4	5	6	6	5
	Cigarette use	4	5	6	6	5
No. of states contributing to control group	E-cigarette use	31	32	37	37	23
	Cigarette use	33	34	38	37	23

[a] Cells marked as "Not Available" represent states and years for which data on either combustible cigarette or e-cigarette use were excluded from the analysis. The absence of data may be due to one or more of the following reasons: (1) The relevant questions were not included in the survey for a specific state or year (2) Data could not be obtained from the state through the requisition process (3) Sampling and other data collection inconsistencies were identified.
*Indicates flavor policy states
**Indicates flavor policy states with mint and/or menthol exemptions

© 2025 Cheng D et al. *JAMA Network Open*.

eTable 2: Availability of E-Cigarette (EC) and Cigarette Use Data in Behavioral Risk Factor Surveillance System[a,b]

State	Question	2015	2016	2017	2018	2019	2020	2021	2022	2023
National BRFSS	E-cigarette use	State-Added	Core	Core	Optional	State-Added	Optional	Core	Core	Core
	Cigarette use	Core	Core	Core	Core	Core	Core	Core	Core	Core
Alabama	E-cigarette use	Not available	Core	Core	Not available	Not available	Optional	Core	Core	Core
	Cigarette use	Core	Core	Core	Core	Core	Core	Core	Core	Core
Alaska	E-cigarette use	State-Added	Core	Core	Optional	State-Added	Optional	Core	Core	Core
	Cigarette use	Core	Core	Core	Core	Core	Core	Core	Core	Core
Arizona	E-cigarette use	State-Added	Core	Core	Not available	Not available	Not available	Core	Core	Core
	Cigarette use	Core	Core	Core	Core	Core	Core	Core	Core	Core
Arkansas	E-cigarette use	Not available	Core	Core	Optional	Not available	Optional	Core	Core	Core
	Cigarette use	Core	Core	Core	Core	Core	Core	Core	Core	Core
California	E-cigarette use	State-Added	Core	Core	State-Added	State-Added	State-Added	Core	Core	Core[c]
	Cigarette use	Core	Core	Core	Core	Core	Core	Core	Core	Core[c]
Colorado	E-cigarette use	Not available	Core	Core	Optional	Not available	Not available	Core	Core	Core
	Cigarette use	Core	Core	Core	Core	Core	Core	Core	Core	Core
Connecticut	E-cigarette use	State-Added	Core	Core	Optional	State-Added	Optional	Core	Core	Core
	Cigarette use	Core	Core	Core	Core	Core	Core	Core	Core	Core
Delaware	E-cigarette use	State Added	Core	Core	Optional	State-Added	Optional	Core	Core	Core
	Cigarette use	Core	Core	Core	Core	Core	Core	Core	Core	Core
D.C.	E-cigarette use	Not available	Core	Core	State-Added	State-Added	State-Added	Core	Core	Core[c]
	Cigarette use	Core	Core	Core	Core	Core	Core	Core	Core	Core[c]
Florida	E-cigarette use	State-Added	Core	Core	Optional	State-Added	Optional	State-Added	Core	Core
	Cigarette use	Core	Core	Core	Core	Core	Core	State-Added	Core	Core
Georgia	E-cigarette use	State-Added	Core	Core	Optional	State-Added	Optional	Core	Core	Core
	Cigarette use	Core	Core	Core	Core	Core	Core	Core	Core	Core
Hawaii	E-cigarette use	Not available	Core	Core	Optional	Not available	Optional	Core	Core	Core
	Cigarette use	Core	Core	Core	Core	Core	Core	Core	Core	Core
Idaho	E-cigarette use	State-Added	Core	Core	Optional	State-Added	Optional	Core	Core	Core
	Cigarette use	Core	Core	Core	Core	Core	Core	Core	Core	Core

© 2025 Cheng D et al. *JAMA Network Open*.

State	Question	2015	2016	2017	2018	2019	2020	2021	2022	2023
Illinois	E-cigarette use	Not available	Core	Core	State-Added	Not available	Optional	Core	Core	Core
	Cigarette use	Core	Core	Core	Core	Core	Core	Core	Core	Core
Indiana	E-cigarette use	Not available	Core	Core	Optional	Not available	Optional	Core	Core	Core
	Cigarette use	Core	Core	Core	Core	Core	Core	Core	Core	Core
Iowa	E-cigarette use	Not available	Core	Core	Optional	Not available	Not available	Core	Core	Core
	Cigarette use	Core	Core	Core	Core	Core	Core	Core	Core	Core
Kansas	E-cigarette use	State-Added	Core	Core	Optional	State-Added	Optional	Core	Core	Core
	Cigarette use	Core	Core	Core	Core	Core	Core	Core	Core	Core
Kentucky	E-cigarette use	Not available	Core	Core	Not available	Not available	Optional	Core	Core	Not available
	Cigarette use	Core	Core	Core	Core	Core	Core	Core	Core	Not available
Louisiana	E-cigarette use	Not available	Core	Core	Optional	Not available	Not available	Core	Core	Core
	Cigarette use	Core	Core	Core	Core	Core	Core	Core	Core	Core
Maine	E-cigarette use	State-Added	Core	Core	Optional	State-Added	Optional	Core	Core	Core
	Cigarette use	Core	Core	Core	Core	Core	Core	Core	Core	Core
**Maryland****	E-cigarette use	Not available	Core	Core	Optional	State-Added	Optional	Core	Core	Core
	Cigarette use	Core	Core	Core	Core	Core	Core	Core	Core	Core
**Massachusetts***	E-cigarette use	State-Added	Core	Core	Optional	State-Added	Optional	Core	Core	Core
	Cigarette use	Core	Core	Core	Core	Core	Core	Core	Core	Core
Michigan	E-cigarette use	Not available	Core	Core	Optional	State-Added	Optional	Core	Core	Core
	Cigarette use	Core	Core	Core	Core	Core	Core	Core	Core	Core
Minnesota	E-cigarette use	Not available	Core	Core	Optional	Not available	Optional	Core	Core	Core
	Cigarette use	Core	Core	Core	Core	Core	Core	Core	Core	Core
Mississippi	E-cigarette use	Not available	Core	Core	Optional	Not available	Optional	Core	Core	Core
	Cigarette use	Core	Core	Core	Core	Core	Core	Core	Core	Core
Missouri	E-cigarette use	State-Added	Core	Core	Optional	State-Added	Optional	Core	Core	Core
	Cigarette use	Core	Core	Core	Core	Core	Core	Core	Core	Core
Montana	E-cigarette use	State-Added	Core	Core	Optional	State-Added	Optional	Core	Core	Core
	Cigarette use	Core	Core	Core	Core	Core	Core	Core	Core	Core
Nebraska	E-cigarette use	Not available	Core	Core	Optional	State-Added	Optional	Core	Core	Core
	Cigarette use	Core	Core	Core	Core	Core	Core	Core	Core	Core

© 2025 Cheng D et al. *JAMA Network Open*.

State	Question	2015	2016	2017	2018	2019	2020	2021	2022	2023
Nevada	E-cigarette use	Not available	Core	Core	Not available	Not available	Optional	Core	Core	Core
	Cigarette use	Core	Core	Core	Core	Core	Core	Core	Core	Core
New Hampshire	E-cigarette use	State-Added	Core	Core	Optional	State-Added	Optional	Core	Core	Core
	Cigarette use	Core	Core	Core	Core	Core	Core	Core	Core	Core
**New Jersey***	E-cigarette use	Not available	Core	Core	Not available	Not available	Optional	Core	Core	Core
	Cigarette use	Core	Core	Core	Core	Not available	Core	Core	Core	Core
New Mexico	E-cigarette use	Not available	Core	Core	Not available	Not available	Optional	Core	Core	Core
	Cigarette use	Core	Core	Core	Core	Core	Core	Core	Core	Core
**New York***	E-cigarette use	Not available	Core	Core	Optional	State-Added	Optional	Core	Core	Core
	Cigarette use	Core	Core	Core	Core	Core	Core	Core	Core	Core
North Carolina	E-cigarette use	State-Added	Core	Core	Optional	State-Added	Optional	Core	Core	Core
	Cigarette use	Core	Core	Core	Core	Core	Core	Core	Core	Core
North Dakota	E-cigarette use	Not available	Core	Core	Optional	Not available	Optional	Core	Core	Core
	Cigarette use	Core	Core	Core	Core	Core	Core	Core	Core	Core
Ohio	E-cigarette use	Not available	Core	Core	Optional	Not available	Optional	Core	Core	Core
	Cigarette use	Core	Core	Core	Core	Core	Core	Core	Core	Core
Oklahoma	E-cigarette use	Not available	Core	Core	Not available	Not available	Not available	Core	Core	Core
	Cigarette use	Core	Core	Core	Core	Core	Core	Core	Core	Core
Oregon	E-cigarette use	State-Added	Core	Core	Optional	State-Added	Optional	Core	Core	Core
	Cigarette use	Core	Core	Core	Core	Core	Core	Core	Core	Core
Pennsylvania	E-cigarette use	Not available	Core	Core	Not available	Not available	Optional	Core	Core	Not available
	Cigarette use	Core	Core	Core	Core	Core	Core	Core	Core	Not available
**Rhode Island***	E-cigarette use	State-Added	Core	Core	Optional	State-Added	Optional	Core	Core	Core
	Cigarette use	Core	Core	Core	Core	Core	Core	Core	Core	Core
South Carolina	E-cigarette use	Not available	Core	Core	Not available	Not available	Not available	Core	Core	Core
	Cigarette use	Core	Core	Core	Core	Core	Core	Core	Core	Core
South Dakota	E-cigarette use	State-Added	Core	Core	Optional	State-Added	Optional	Core	Core	Core
	Cigarette use	Core	Core	Core	Core	Core	Core	Core	Core	Core

© 2025 Cheng D et al. *JAMA Network Open*.

State	Question	2015	2016	2017	2018	2019	2020	2021	2022	2023
Tennessee	E-cigarette use	Not available	Core	Core	Optional	State-Added	Optional	Core	Core	Core
	Cigarette use	Core	Core	Core	Core	Core	Core	Core	Core	Core
Texas	E-cigarette use	State-Added	Core	Core	Optional	State-Added	Optional	Core	Core	Core
	Cigarette use	Core	Core	Core	Core	Core	Core	Core	Core	Core
**Utah****	E-cigarette use	State-Added	Core	Core	Optional	State-Added	Optional	Core	Core	Core
	Cigarette use	Core	Core	Core	Core	Core	Core	Core	Core	Core
Vermont	E-cigarette use	Not available	Core	Core	Not available	Not available	Optional	Core	Core	Core
	Cigarette use	Core	Core	Core	Core	Core	Core	Core	Core	Core
Virginia	E-cigarette use	Not available	Core	Core	Optional	State-Added	Optional	Core	Core	Core
	Cigarette use	Core	Core	Core	Core	Core	Core	Core	Core	Core
Washington	E-cigarette use	State-Added	Core	Core	Not available	Not available	Optional	Core	Core	Core
	Cigarette use	Core	Core	Core	Core	Core	Core	Core	Core	Core
West Virginia	E-cigarette use	Not available	Core	Core	Not available	Not available	Optional	Core	Core	Core
	Cigarette use	Core	Core	Core	Core	Core	Core	Core	Core	Core
Wisconsin	E-cigarette use	Not available	Core	Core	Optional	State-Added	State-Added	Core	Core	Core
	Cigarette use	Core	Core	Core	Core	Core	Core	Core	Core	Core
Wyoming	E-cigarette use	Not available	Core	Core	Optional	State-Added	Optional	Core	Core	Core
	Cigarette use	Core	Core	Core	Core	Core	Core	Core	Core	Core
No. of available data state-years	E-cigarette use	21	51	51	39	28	45	51	51	49
	Cigarette use	51	51	51	51	50	51	51	51	49
No. of states contributing to policy group	E-cigarette use	3	6	6	5	5	6	6	6	6
	Cigarette use	6	6	6	6	5	6	6	6	6
No. of states contributing to control group	E-cigarette use	18	45	45	34	23	39	45	45	41
	Cigarette use	45	45	45	45	45	45	45	45	41

[a] Cells marked as "Not Available" represent states and years for which data on either combustible cigarette or e-cigarette use were excluded from the analysis. The absence of data may be due to one or more of the following reasons: (1) The relevant questions were not included in the survey for a specific state or year (2) Data could not be obtained from the state through the requisition process (3) Sampling and other data collection inconsistencies were identified (4) Data files were unreadable/incompatible.

[b] State-years denoted as “Core” were those where questions pertaining to e-cigarette and/or cigarette use were mandated to be included in all states’ BRFSS questionnaires as a core survey component developed by the CDC. State-years denoted as “Optional” were those where states elected to include questions pertaining to e-cigarette use as an optional module offered and developed by the CDC. State-years denoted as “State-Added” were those where a state elected to include their own set of questions pertaining to e-cigarette use, outside of the CDC’s review. Data for the “State-Added” state-years were obtained through requests with individual states.

© 2025 Cheng D et al. *JAMA Network Open*.

[c] BRFSS data in 2023 for California and D.C. were obtained but were ultimately excluded from both the policy and control groups for 2023 estimates because these states enacted their own flavor policies at the end of 2022.
*Indicates flavor policy states
**Indicates flavor policy states with mint and/or menthol exemptions

© 2025 Cheng D et al. *JAMA Network Open*.

eTable 3: Summary of state-level aggregate characteristics among policy and control states by age group

	Youth		*Young Adult (Age 18-24)*		*Adult (Age ≥25)*	
**Characteristics from 2019 Survey**	**Policy States**	**Control States**	**Policy States**	**Control States**	**Policy States**	**Control States**
States contributing data, N						
EC	6	37	5	23	5	23
Cigarette	6	38	5	45	5	45
Respondents contributing data (unweighted), N						
EC	54,143	107,692	2,553	8,324	45,308	139,623
Cigarette	57,590	123,051	3,175	20,394	51,202	320,175
Mean age in years, mean	16.0	16.0	21.0	20.9	51.1	51.7
% in each grade, mean						
Freshman	26.4	26.8	NA	NA	NA	NA
Sophomore	25.5	25.6	NA	NA	NA	NA
Junior	24.2	24.1	NA	NA	NA	NA
Senior	23.8	23.6	NA	NA	NA	NA
% male, mean	50.8	51.2	50.6	51.9	48	48.6
% by race/ethnicity, mean						
Black only	13.7	13.0	11.9	10.7	11.1	10.5
Hispanic	21.0	16.9	16.3	16.4	13.2	9.8
Multiracial	2.9	3.8	2.7	2.7	1.0	1.6
White only	55.3	58.4	58.5	61.1	67.7	72.0
Additional race categories[a]	7.1	7.9	10.7	9.1	6.9	6.2
% current substance use, mean						
Current EC use	24.1	24.6	18.0	17.4	4.2	4.3
Current cigarette use	4.0	6.5	6.7	12.3	12.4	17.4
Current alcohol use	23.7	26.0	50.9	53.2	52.5	53.4
Current marijuana use	19.2	18.9	22.2	27.3	10.0	13.5

EC=e-cigarette.

[a] "American Indian or Alaska Native,", "Asian", "Pacific Islander," "Other"

© 2025 Cheng D et al. *JAMA Network Open*.

	Youth		Young Adult (18 – 24)		Adult (Age ≥25)	
**Characteristics from 2021 Survey**	**Policy States**	**Control States**	**Policy States**	**Control States**	**Policy States**	**Control States**
States contributing data, N						
EC	6	37	6	45	6	45
Cigarette	6	37	6	45	6	45
Respondents contributing data (unweighted), N						
EC	45,335	93,312	4,881	19,514	75,814	315,029
Cigarette	47,171	88,454	4,886	19,551	75,545	314,206
Mean age in years, mean	15.7	15.8	20.9	20.9	51.5	52.0
% in each grade, mean						
Freshman	26.5	27.1	NA	NA	NA	NA
Sophomore	25.3	25.5	NA	NA	NA	NA
Junior	24.3	24.1	NA	NA	NA	NA
Senior	23.9	23.3	NA	NA	NA	NA
% male, mean	50.2	50.9	50.7	51.6	48.0	48.7
% by race/ethnicity, mean						
Black only	13.8	13.5	11.7	11.1	11.5	10.5
Hispanic	23.4	17.1	19.9	17.3	14.3	10.1
Multiracial	3.4	4.4	2.1	2.9	1.1	1.7
White only	52.3	58	55.5	60	65.3	71.4
Additional race categories[a]	7.0	7.0	10.8	8.7	7.8	6.4
% current substance use, mean						
Current EC use	16.1	18.5	16.1	19.9	4.1	5.1
Current cigarette use	3.4	4.3	5.6	7.5	11.2	15.9
Current alcohol use	18.9	22.0	46.7	50.0	52.6	52.9
Current marijuana use	14.4	14.7	21.3	24.9	10.0	12.9

EC=e-cigarette.
[a] "American Indian or Alaska Native," "Pacific Islander," "Other"

© 2025 Cheng D et al. *JAMA Network Open*.

eTable 4: Summary of survey non-response to e-cigarette and cigarette questions by age group

				2015	2016	2017	2018	2019	2020	2021	2022	2023
**Youth**	Policy States	E-cigarettes	N states	4		5		6		6		5
			N individuals	73012		69853		58710		47752		43154
			% missing	3.49		8.95		7.78		5.06		1.33
		Cigarettes	N states	4		5		6		6		5
			N individuals	73012		69853		58710		47752		43154
			% missing	4.71		1.38		1.91		1.22		1
	Control States	E-cigarettes	N states	31		32		37		37		23
			N individuals	127501		114560		121559		100097		70654
			% missing	1.68		8.56		11.41		6.78		6.73
		Cigarettes	N states	33		34		38		37		23
			N individuals	136258		123156		127262		91012		70654
			% missing	3.92		3.19		3.31		2.81		3.07
**Young adult**	Policy States	E-cigarettes	N states	3	6	6	5	5	6	6	6	6
			N individuals	1902	4654	3506	4668	3348	4270	5200	4676	4687
			% missing	43.69	6.06	5.79	12.55	23.75	27.33	6.13	9.84	5.59
		Cigarettes	N states	6	6	6	6	5	6	6	6	6
			N individuals	3488	4654	3506	4876	3348	4270	5200	4676	4687
			% missing	5.39	5.93	5.16	4.49	5.17	6.18	6.04	9.35	5.16
	Control States	E-cigarettes	N states	18	45	45	34	23	39	45	45	43
			N individuals	9108	21371	22848	16791	11509	17782	20627	21661	21100
			% missing	40.1	4.56	7.85	26.02	27.67	20.13	5.4	7.43	5.29
		Cigarettes	N states	45	45	45	45	45	45	45	45	43
			N individuals	20261	21371	22848	21056	21404	20793	20627	21661	21100
			% missing	4.74	4.49	7.29	6.18	4.72	5.09	5.22	7.25	4.94

© 2025 Cheng D et al. *JAMA Network Open*.

				2015	2016	2017	2018	2019	2020	2021	2022	2023
**Adult**	Policy States	E-cigarettes	N states	3	6	6	5	5	6	6	6	6
			N individuals	24999	80521	56845	71484	53936	59653	81400	64499	65708
			% missing	33.78	5.03	4.92	6.18	16	24.29	6.86	9.99	6.39
		Cigarettes	N states	6	6	6	6	5	6	6	6	6
			N individuals	59833	80521	56845	74366	53936	59653	81400	64499	65708
			% missing	4.65	4.91	4.26	4.22	5.07	6.78	7.19	9.74	6.3
	Control States	E-cigarettes	N states	18	45	45	34	23	39	45	45	43
			N individuals	157675	371119	369992	260676	176535	269101	332354	344889	333611
			% missing	35.73	3.72	6.74	19	20.91	14.48	5.21	7.76	5.22
		Cigarettes	N states	45	45	45	45	45	45	45	45	43
			N individuals	354421	371119	369992	336459	335221	310051	332354	344889	333611
			% missing	3.98	3.9	6.36	5.3	4.49	5.02	5.46	7.76	5.21

N states: number of states, among the policy and control states, where respective questions on ECs and cigarette use are fielded.
N individuals: count of total respondents to the corresponding survey among states fielding questions on EC or cigarette use.
% missing: proportion of total respondents with missing, refused or "don't know" responses to questions on EC or cigarette use

© 2025 Cheng D et al. *JAMA Network Open*.

eTable 5: Association of flavor restriction policy with current e-cigarette and cigarette use by age group and specific states (numerical estimates)

		E-cigarette use		Cigarette use	
	Year	ATT (95% CI)	p-value	ATT (95% CI)	p-value
Youth (Grade 9-12, YRBS)					
MA, NY, NJ, RI, MD, UT	2021	-0.019 (-0.074 to 0.035)	0.43	0.018 (0.007 to 0.029)	0.007
MA, NY, NJ, RI		-0.039 (-0.105 to 0.028)	0.18	0.019 (0.002 to 0.036)	0.04
Massachusetts		-0.09 (-0.106 to -0.073)	<0.001	0.009 (0.003 to 0.014)	0.004
New York		-0.006 (-0.023 to 0.01)	0.45	0.032 (0.026 to 0.037)	<0.001
Rhode Island		-0.06 (-0.077 to -0.044)	<0.001	0.012 (0.007 to 0.018)	<0.001
Maryland		-0.022 (-0.039 to -0.006)	0.01	0.01 (0.004 to 0.015)	<0.001
Utah		0.061 (0.044 to 0.077)	<0.001	0.021 (0.015 to 0.026)	<0.001
MA, NY, NJ, RI, MD, UT	2023	-0.027 (-0.07 to 0.017)	0.19	0.006 (-0.004 to 0.017)	0.19
MA, NY, NJ, RI		-0.039 (-0.087 to 0.008)	0.08	0.006 (-0.011 to 0.023)	0.35
Massachusetts		-0.066 (-0.087 to -0.045)	<0.001	0.006 (-0.002 to 0.014)	0.12
New York		-0.013 (-0.034 to 0.008)	0.22	-0.002 (-0.009 to 0.006)	0.66
Maryland		-0.012 (-0.033 to 0.009)	0.24	0.006 (-0.002 to 0.013)	0.15
Utah		0.034 (0.014 to 0.055)	0.002	0.011 (0.003 to 0.018)	0.01
Young adult (Age 18-24, BRFSS)					
MA, NY, NJ, RI, MD, UT	2020	-0.041 (-0.093 to 0.012)	0.11	0.014 (-0.001 to 0.028)	0.06
MA, NY, NJ, RI		-0.058 (-0.155 to 0.038)	0.16	0.009 (-0.016 to 0.034)	0.34
Massachusetts		-0.08 (-0.099 to -0.062)	<0.001	0.014 (0.006 to 0.023)	0.001
New York		-0.097 (-0.116 to -0.078)	<0.001	0.004 (-0.004 to 0.012)	0.35
Rhode Island		0.002 (-0.016 to 0.021)	0.79	0.023 (0.015 to 0.032)	<0.001
Maryland		-0.024 (-0.042 to -0.005)	0.01	0.019 (0.011 to 0.028)	<0.001
Utah		-0.002 (-0.021 to 0.016)	0.8	0.02 (0.012 to 0.028)	<0.001
MA, NY, NJ, RI, MD, UT	2021	-0.044 (-0.111 to 0.023)	0.16	0.037 (0.022 to 0.052)	0.001
MA, NY, NJ, RI		-0.049 (-0.185 to 0.087)	0.34	0.036 (0.005 to 0.066)	0.03
Massachusetts		-0.108 (-0.126 to -0.09)	<0.001	0.043 (0.034 to 0.053)	<0.001

© 2025 Cheng D et al. *JAMA Network Open*.

		E-cigarette use		Cigarette use	
	Year	ATT (95% CI)	p-value	ATT (95% CI)	p-value
Young adult (Age 18-24, BRFSS)					
New York	2021	-0.084 (-0.103 to -0.066)	<0.001	0.034 (0.025 to 0.044)	<0.001
Rhode Island		0.047 (0.028 to 0.065)	<0.001	0.051 (0.041 to 0.061)	<0.001
Maryland		-0.017 (-0.036 to 0.001)	0.07	0.035 (0.026 to 0.045)	<0.001
Utah		-0.069 (-0.087 to -0.05)	<0.001	0.036 (0.027 to 0.046)	<0.001
MA, NY, NJ, RI, MD, UT	2022	-0.067 (-0.121 to -0.013)	0.02	0.027 (0.014 to 0.041)	0.003
MA, NY, NJ, RI		-0.076 (-0.189 to 0.038)	0.13	0.024 (0.006 to 0.042)	0.02
Massachusetts		-0.123 (-0.144 to -0.103)	<0.001	0.031 (0.022 to 0.041)	<0.001
New York		-0.08 (-0.1 to -0.06)	<0.001	0.029 (0.02 to 0.039)	<0.001
Rhode Island		0 (-0.021 to 0.02)	0.97	0.02 (0.01 to 0.029)	<0.001
Maryland		-0.055 (-0.075 to -0.035)	<0.001	0.023 (0.014 to 0.033)	<0.001
Utah		-0.063 (-0.083 to -0.043)	<0.001	0.037 (0.028 to 0.047)	<0.001
MA, NY, NJ, RI, MD, UT	2023	-0.06 (-0.124 to 0.003)	0.06	0.032 (0.009 to 0.055)	0.01
MA, NY, NJ, RI		-0.064 (-0.186 to 0.059)	0.2	0.028 (-0.006 to 0.062)	0.08
Massachusetts		-0.128 (-0.152 to -0.104)	<0.001	0.02 (0.01 to 0.03)	<0.001
New York		-0.072 (-0.096 to -0.048)	<0.001	0.022 (0.012 to 0.032)	<0.001
Rhode Island		0.012 (-0.012 to 0.036)	0.3	0.014 (0.004 to 0.024)	0.006
Maryland		-0.03 (-0.053 to -0.006)	0.02	0.039 (0.029 to 0.049)	<0.001
Utah		-0.094 (-0.118 to -0.071)	<0.001	0.036 (0.026 to 0.046)	<0.001
Adult (Age ≥25, BRFSS)					
MA, NY, NJ, RI, MD, UT	2020	-0.001 (-0.012 to 0.009)	0.81	0 (-0.012 to 0.013)	0.96
MA, NY, NJ, RI		-0.004 (-0.023 to 0.015)	0.54	-0.001 (-0.013 to 0.011)	0.75
Massachusetts		-0.014 (-0.018 to -0.011)	<0.001	-0.004 (-0.007 to 0)	0.03
New York		0.005 (0.001 to 0.009)	0.01	0.002 (-0.002 to 0.005)	0.32
Rhode Island		-0.009 (-0.013 to -0.006)	<0.001	0.008 (0.005 to 0.011)	<0.001
Maryland		0.001 (-0.003 to 0.005)	0.72	-0.013 (-0.016 to -0.01)	<0.001
Utah		0.01 (0.006 to 0.013)	<0.001	0.011 (0.007 to 0.014)	<0.001

© 2025 Cheng D et al. *JAMA Network Open*.

	Year	E-cigarette use		Cigarette use	
		**ATT (95% CI)**	**p-value**	**ATT (95% CI)**	**p-value**
Adult (Age ≥25, BRFSS)					
MA, NY, NJ, RI, MD, UT	2021	-0.009 (-0.02 to 0.003)	0.11	0.002 (-0.01 to 0.014)	0.65
MA, NY, NJ, RI		-0.011 (-0.033 to 0.01)	0.2	0.002 (-0.008 to 0.013)	0.56
Massachusetts		-0.023 (-0.028 to -0.019)	<0.001	-0.001 (-0.005 to 0.002)	0.45
New York		0 (-0.004 to 0.005)	0.84	0.01 (0.006 to 0.013)	<0.001
Rhode Island		-0.016 (-0.021 to -0.012)	<0.001	0.003 (0 to 0.007)	0.05
Maryland		-0.01 (-0.014 to -0.006)	<0.001	-0.013 (-0.016 to -0.009)	<0.001
Utah		0.001 (-0.003 to 0.006)	0.53	0.009 (0.005 to 0.012)	<0.001
MA, NY, NJ, RI, MD, UT	2022	-0.01 (-0.023 to 0.003)	0.12	0.006 (-0.005 to 0.018)	0.22
MA, NY, NJ, RI		-0.009 (-0.036 to 0.017)	0.36	0.007 (-0.003 to 0.016)	0.1
Massachusetts		-0.023 (-0.028 to -0.018)	<0.001	0.006 (0.002 to 0.01)	0.002
New York		0.01 (0.005 to 0.015)	<0.001	0.01 (0.006 to 0.014)	<0.001
Rhode Island		-0.015 (-0.02 to -0.01)	<0.001	0.009 (0.005 to 0.013)	<0.001
Maryland		-0.016 (-0.021 to -0.011)	<0.001	-0.009 (-0.013 to -0.005)	<0.001
Utah		-0.007 (-0.012 to -0.002)	0.01	0.011 (0.007 to 0.015)	<0.001
MA, NY, NJ, RI, MD, UT	2023	-0.012 (-0.02 to -0.004)	0.009	0.006 (-0.008 to 0.021)	0.34
MA, NY, NJ, RI		-0.012 (-0.026 to 0.002)	0.07	0.003 (-0.017 to 0.023)	0.67
Massachusetts		-0.015 (-0.021 to -0.009)	<0.001	0.016 (0.009 to 0.022)	<0.001
New York		-0.006 (-0.012 to 0)	0.03	0.003 (-0.003 to 0.009)	0.35
Rhode Island		-0.016 (-0.021 to -0.01)	<0.001	-0.002 (-0.008 to 0.004)	0.57
Maryland		-0.014 (-0.019 to -0.008)	<0.001	-0.002 (-0.008 to 0.004)	0.5
Utah		-0.011 (-0.017 to -0.006)	<0.001	0.018 (0.012 to 0.024)	<0.001

© 2025 Cheng D et al. *JAMA Network Open*.

eTable 6: Association of flavor restriction policy with current cigarette use by e-cigarette (EC) use history

	Among Current EC Users		*Among Former EC Users*		*Among Never EC Users*	
**Year**	**ATT (95% CI)**	**p-value**	**ATT (95% CI)**	**p-value**	**ATT (95% CI)**	**p-value**
Youths (Grade 9-12, YRBSS)						
2021	0.08 (0.034 to 0.126)	0.005	0.018 (-0.003 to 0.04)	0.08	0 (-0.004 to 0.004)	1.00
2023	0.03 (-0.028 to 0.089)	0.26	0.016 (0.001 to 0.032)	0.04	0 (-0.004 to 0.004)	0.92
Young adults (Age 18-24, BRFSS)						
2020	0.021 (-0.09 to 0.132)	0.66	0.037 (-0.018 to 0.091)	0.15	0.009 (-0.01 to 0.029)	0.29
2021	0.083 (-0.005 to 0.172)	0.06	0.082 (0.016 to 0.149)	0.02	0.028 (-0.014 to 0.069)	0.15
2022	0.026 (-0.076 to 0.128)	0.56	0.077 (0.007 to 0.146)	0.03	0.019 (-0.001 to 0.038)	0.06
2023	0.034 (-0.075 to 0.143)	0.48	0.083 (0.016 to 0.15)	0.02	0.022 (0.003 to 0.04)	0.03
Adults (Age ≥25, BRFSS)						
2020	0.008 (-0.079 to 0.095)	0.83	0.058 (-0.136 to 0.251)	0.50	0.003 (-0.029 to 0.036)	0.81
2021	0.011 (-0.075 to 0.096)	0.77	0.102 (-0.109 to 0.313)	0.29	0.001 (-0.04 to 0.043)	0.94
2022	0.036 (-0.021 to 0.093)	0.18	0.059 (-0.155 to 0.273)	0.53	0.007 (-0.027 to 0.042)	0.63
2023	-0.002 (-0.076 to 0.072)	0.95	0.077 (-0.156 to 0.31)	0.46	0.007 (-0.027 to 0.04)	0.66

ATT=average treatment effect among treated. Estimates based on difference-in-difference analyses between policy (MA, NJ, NY, RI, MD, UT) and control states and prevalence of use in pre-policy (2019) and specified post-policy year.

CIs and p-values based on cluster-robust standard errors clustered by state with small-sample correction.

The young adult and adult prevalence of cigarette use among those formerly and never using e-cigarettes may be biased in 2021 due to a change in the BRFSS questionnaire. Estimates from these data need to be interpreted with caution.

© 2025 Cheng D et al. *JAMA Network Open*.

eTable 7: State-year inclusion of BRFSS and YRBS data in Figure 3 sensitivity analyses[a]

		Balanced[b]		Pre-year 2017[c]		EC taxation[d]		No T21[e]		Require Licensure[f]		EC clean indoor air[g]	
**State**	**Outcome**	BRFSS	YRBS	BRFSS	YRBS	BRFSS	YRBS	BRFSS	YRBS	BRFSS	YRBS	BRFSS	YRBS
**Maryland**	E-cigarette use	B_{19-23}	$Y_{19,21,23}$	$B_{17,20-23}$	$Y_{17,21,23}$	B_{21-23}	$Y_{21,23}$	B_{19}	Y_{19}	B_{19-23}	$Y_{19,21,23}$	--	--
	Cigarette use	B_{19-23}	$Y_{19,21,23}$	$B_{17,20-23}$	$Y_{17,21,23}$	B_{21-23}	$Y_{21,23}$	B_{19}	Y_{19}	B_{19-23}	$Y_{19,21,23}$	--	--
**Massachusetts**	E-cigarette use	B_{19-23}	$Y_{19,21,23}$	$B_{17,20-23}$	$Y_{17,21,23}$	B_{20-23}	$Y_{21,23}$	B_{19-21}	$Y_{19,21}$	B_{20-23}	$Y_{21,23}$	B_{19-23}	$Y_{19,21,23}$
	Cigarette use	B_{19-23}	$Y_{19,21,23}$	$B_{17,20-23}$	$Y_{17,21,23}$	B_{20-23}	$Y_{21,23}$	B_{19-21}	$Y_{19,21}$	B_{20-23}	$Y_{21,23}$	B_{19-23}	$Y_{19,21,23}$
**New Jersey**	E-cigarette use	--	$Y_{19,21,23}$	$B_{17,20-23}$	$Y_{21,23}$	B_{20-23}	$Y_{19,21,23}$	--	--	B_{20-23}	$Y_{21,23}$	B_{20-23}	$Y_{19,21,23}$
	Cigarette use	--	$Y_{19,21,23}$	$B_{17,20-23}$	$Y_{21,23}$	B_{20-23}	$Y_{19,21,23}$	--	--	B_{20-23}	$Y_{21,23}$	B_{20-23}	$Y_{19,21,23}$
**New York**	E-cigarette use	B_{19-23}	$Y_{19,21,23}$	$B_{17,20-23}$	$Y_{17,21,23}$	B_{19-23}	$Y_{19,21,23}$	B_{19}	Y_{19}	B_{20-23}	$Y_{21,23}$	B_{19-23}	$Y_{19,21,23}$
	Cigarette use	B_{19-23}	$Y_{19,21,23}$	$B_{17,20-23}$	$Y_{17,21,23}$	B_{19-23}	$Y_{19,21,23}$	B_{19}	Y_{19}	B_{20-23}	$Y_{21,23}$	B_{19-23}	$Y_{19,21,23}$
**Rhode Island**	E-cigarette use	B_{19-23}	$Y_{19,21}$	$B_{17,20-23}$	$Y_{17,21}$	--	--	B_{19-21}	$Y_{19,21}$	B_{19-23}	$Y_{19,21}$	B_{19-23}	$Y_{19,21}$
	Cigarette use	B_{19-23}	$Y_{19,21}$	$B_{17,20-23}$	$Y_{17,21}$	--	--	B_{19-21}	$Y_{19,21}$	B_{19-23}	$Y_{19,21}$	B_{19-23}	$Y_{19,21}$
**Utah**	E-cigarette use	B_{19-23}	$Y_{19,21,23}$	$B_{17,20-23}$	$Y_{17,21,23}$	B_{20-23}	$Y_{21,23}$	$B_{19,20}$	Y_{19}	B_{19-23}	$Y_{19,21,23}$	B_{19-23}	$Y_{19,21,23}$
	Cigarette use	B_{19-23}	$Y_{19,21,23}$	$B_{17,20-23}$	$Y_{17,21,23}$	B_{20-23}	$Y_{21,23}$	$B_{19,20}$	Y_{19}	B_{19-23}	$Y_{19,21,23}$	B_{19-23}	$Y_{19,21,23}$
Alabama	E-cigarette use	--	$Y_{19,21}$	$B_{17,20-23}$	Y_{21}	--	--	B_{20-21}	$Y_{19,21}$	B_{20-23}	Y_{21}	--	--
	Cigarette use	B_{19-23}	$Y_{19,21}$	$B_{17,20-23}$	Y_{21}	--	--	B_{19-21}	$Y_{19,21}$	B_{20-23}	Y_{21}	--	--
Alaska	E-cigarette use	B_{19-23}	$Y_{19,23}$	$B_{17,20-23}$	$Y_{17,23}$	--	--	B_{19-23}	$Y_{19,23}$	B_{19-23}	$Y_{19,23}$	--	--
	Cigarette use	B_{19-23}	$Y_{19,23}$	$B_{17,20-23}$	$Y_{17,23}$	--	--	B_{19-23}	$Y_{19,23}$	B_{19-23}	$Y_{19,23}$	--	--
Arizona	E-cigarette use	--	$Y_{19,21}$	$B_{17,21-23}$	$Y_{17,21}$	--	--	B_{21-23}	$Y_{19,21}$	--	--	--	--
	Cigarette use	B_{19-23}	$Y_{19,21}$	$B_{17,20-23}$	$Y_{17,21}$	--	--	B_{19-23}	$Y_{19,21}$	--	--	--	--
Arkansas	E-cigarette use	--	$Y_{19,21,23}$	$B_{17,20-23}$	$Y_{17,21,23}$	--	--	B_{20-21}	$Y_{19,21}$	B_{20-23}	$Y_{19,21,23}$	--	--
	Cigarette use	B_{19-23}	$Y_{19,21,23}$	$B_{17,20-23}$	$Y_{17,21,23}$	--	--	B_{19-21}	$Y_{19,21}$	B_{19-23}	$Y_{19,21,23}$	--	--
California	E-cigarette use	B_{19-22}	--	$B_{17,20-22}$	Y_{17}	B_{19-22}	Y_{19}	--	--	B_{19-22}	Y_{19}	B_{19-22}	Y_{19}
	Cigarette use	B_{19-22}	--	$B_{17,20-22}$	Y_{17}	B_{19-22}	--	--	--	B_{19-22}	--	B_{19-22}	--
Colorado	E-cigarette use	--	$Y_{19,21}$	$B_{17,21-23}$	$Y_{17,21}$	B_{21-23}	Y_{21}	--	Y_{19}	$B_{22,23}$	--	B_{21-23}	Y_{21}
	Cigarette use	B_{19-23}	$Y_{19,21}$	$B_{17,20-23}$	$Y_{17,21}$	B_{21-23}	Y_{21}	$B_{19,20}$	Y_{19}	$B_{22,23}$	--	B_{20-23}	Y_{21}
Connecticut	E-cigarette use	B_{19-23}	$Y_{19,21}$	$B_{17,20-23}$	Y_{21}	B_{19-23}	$Y_{19,21}$	B_{19}	Y_{19}	B_{19-23}	$Y_{19,21}$	$B_{22,23}$	--
	Cigarette use	B_{19-23}	$Y_{19,21}$	$B_{17,20-23}$	$Y_{17,21}$	B_{19-23}	$Y_{19,21}$	B_{19}	Y_{19}	B_{19-23}	$Y_{19,21}$	$B_{22,23}$	--
Delaware	E-cigarette use	B_{19-23}	--	$B_{17,20-23}$	$Y_{17,21}$	B_{19-23}	Y_{21}	B_{19}	--	--	--	B_{19-23}	Y_{21}
	Cigarette use	B_{19-23}	--	$B_{17,20-23}$	$Y_{17,21}$	B_{19-23}	Y_{21}	B_{19}	--	--	--	B_{19-23}	Y_{21}
D.C.	E-cigarette use	B_{19-22}	--	$B_{17,20-22}$	--	B_{19-22}	--	--	--	B_{19-22}	--	B_{19-22}	--
	Cigarette use	B_{19-22}	--	$B_{17,20-22}$	--	B_{19-22}	--	--	--	B_{19-22}	--	B_{19-22}	--
Florida	E-cigarette use	B_{19-23}	--	$B_{17,20-23}$	--	--	--	B_{19-21}	--	$B_{22,23}$	--	--	--
	Cigarette use	B_{19-23}	$Y_{19,21}$	$B_{17,20-23}$	$Y_{17,21}$	--	--	B_{19-21}	$Y_{19,21}$	$B_{22,23}$	--	--	--
Georgia	E-cigarette use	B_{19-23}	$Y_{19,21}$	$B_{17,20-23}$	Y_{21}	B_{21-23}	Y_{21}	$B_{19,20}$	Y_{19}	B_{21-23}	Y_{21}	--	--
	Cigarette use	B_{19-23}	$Y_{19,21}$	$B_{17,20-23}$	Y_{21}	B_{21-23}	Y_{21}	$B_{19,20}$	Y_{19}	B_{21-23}	Y_{21}	--	--

© 2025 Cheng D et al. *JAMA Network Open*.

State	Outcome	Balanced[b]		Pre-year 2017[c]		EC taxation[d]		No T21[e]		Require Licensure[f]		EC clean indoor air[g]	
Hawaii	E-cigarette use	--	$Y_{19,21,23}$	$B_{17,20-23}$	$Y_{17,21,23}$	--	--	--	--	B_{20-23}	$Y_{19,21,23}$	B_{20-23}	$Y_{19,21,23}$
	Cigarette use	B_{19-23}	$Y_{19,21,23}$	$B_{17,20-23}$	$Y_{17,21,23}$	--	--	--	--	B_{19-23}	$Y_{19,21,23}$	B_{19-23}	$Y_{19,21,23}$
Idaho	E-cigarette use	B_{19-23}	$Y_{19,21}$	$B_{17,20-23}$	$Y_{17,21}$	--	--	B_{19-22}	$Y_{19,21}$	B_{21-23}	Y_{21}	--	--
	Cigarette use	B_{19-23}	$Y_{19,21}$	$B_{17,20-23}$	$Y_{17,21}$	--	--	B_{19-22}	$Y_{19,21}$	B_{21-23}	Y_{21}	--	--
Illinois	E-cigarette use	--	$Y_{19,21,23}$	$B_{17,20-23}$	$Y_{17,21,23}$	B_{20-23}	$Y_{19,21,23}$	--	Y_{19}	--	--	--	--
	Cigarette use	B_{19-23}	$Y_{19,21,23}$	$B_{17,20-23}$	$Y_{17,21,23}$	B_{19-23}	$Y_{19,21,23}$	B_{19}	Y_{19}	--	--	--	--
Indiana	E-cigarette use	--	--	$B_{17,20-23}$	$Y_{21,23}$	$B_{22,23}$	Y_{23}	B_{20}	--	B_{20-23}	$Y_{21,23}$	--	--
	Cigarette use	B_{19-23}	--	$B_{17,20-23}$	$Y_{21,23}$	$B_{22,23}$	Y_{23}	$B_{19,20}$	--	B_{19-23}	$Y_{21,23}$	--	--
Iowa	E-cigarette use	--	$Y_{19,21}$	$B_{17,21-23}$	$Y_{17,21}$	--	--	--	Y_{19}	B_{21-23}	$Y_{19,21}$	--	--
	Cigarette use	B_{19-23}	$Y_{19,21}$	$B_{17,20-23}$	$Y_{17,21}$	--	--	B_{19}	Y_{19}	B_{19-23}	$Y_{19,21}$	--	--
Kansas	E-cigarette use	B_{19-23}	$Y_{19,21}$	$B_{17,20-23}$	$Y_{17,21}$	B_{19-23}	$Y_{19,21}$	B_{19-23}	$Y_{19,21}$	B_{19-23}	$Y_{19,21}$	--	--
	Cigarette use	B_{19-23}	$Y_{19,21}$	$B_{17,20-23}$	$Y_{17,21}$	B_{19-23}	$Y_{19,21}$	B_{19-23}	$Y_{19,21}$	B_{19-23}	$Y_{19,21}$	--	--
Kentucky	E-cigarette use	--	$Y_{19,21,23}$	$B_{17,20-22}$	$Y_{17,21,23}$	B_{20-22}	$Y_{21,23}$	--	Y_{19}	--	--	--	--
	Cigarette use	B_{19-22}	$Y_{19,21,23}$	$B_{17,20-22}$	$Y_{17,21,23}$	B_{20-22}	$Y_{21,23}$	B_{19}	Y_{19}	--	--	--	--
Louisiana	E-cigarette use	--	$Y_{19,21}$	$B_{17,21-23}$	$Y_{17,21}$	B_{21-23}	$Y_{19,21}$	B_{21}	$Y_{19,21}$	B_{21-23}	$Y_{19,21}$	--	--
	Cigarette use	B_{19-23}	$Y_{19,21}$	$B_{17,20-23}$	$Y_{17,21}$	B_{19-23}	$Y_{19,21}$	B_{19-21}	$Y_{19,21}$	B_{19-23}	$Y_{19,21}$	--	--
Maine	E-cigarette	B_{19-23}	$Y_{19,21}$	$B_{17,20-23}$	$Y_{17,21}$	B_{20-23}	Y_{21}	B_{19-21}	$Y_{19,21}$	B_{19-23}	$Y_{19,21}$	--	--
	Cigarette use	B_{19-23}	$Y_{19,21}$	$B_{17,20-23}$	$Y_{17,21}$	B_{20-23}	Y_{21}	B_{19-21}	$Y_{19,21}$	B_{19-23}	$Y_{19,21}$	--	--
Michigan	E-cigarette use	B_{19-23}	$Y_{19,21,23}$	$B_{17,20-23}$	$Y_{17,21,23}$	--	--	B_{19-22}	$Y_{19,21}$	--	--	--	--
	Cigarette use	B_{19-23}	$Y_{19,21,23}$	$B_{17,20-23}$	$Y_{17,21,23}$	--	--	B_{19-22}	$Y_{19,21}$	--	--	--	--
Minnesota	E-cigarette use	--	--	$B_{17,20-23}$	--	B_{20-23}	--	B_{20}	--	B_{20-23}	--	B_{20-23}	--
	Cigarette use	B_{19-23}	--	$B_{17,20-23}$	--	B_{19-23}	--	$B_{19,20}$	--	B_{19-23}	--	B_{20-23}	--
Mississippi	E-cigarette use	--	$Y_{19,21,23}$	$B_{17,20-23}$	$Y_{21,23}$	--	--	B_{20}	Y_{19}	--	--	--	--
	Cigarette use	B_{19-23}	$Y_{19,21,23}$	$B_{17,20-23}$	$Y_{21,23}$	--	--	$B_{19,20}$	Y_{19}	--	--	--	--
Missouri	E-cigarette use	B_{19-23}	$Y_{19,21,23}$	$B_{17,20-23}$	$Y_{17,21,23}$	--	--	B_{19-23}	$Y_{19,21,23}$	--	--	--	--
	Cigarette use	B_{19-23}	$Y_{19,21,23}$	$B_{17,20-23}$	$Y_{17,21,23}$	--	--	B_{19-23}	$Y_{19,21,23}$	--	--	--	--
Montana	E-cigarette use	B_{19-23}	$Y_{19,21,23}$	$B_{17,20-23}$	$Y_{17,21,23}$	--	--	B_{19-23}	$Y_{19,21,23}$	B_{19-23}	$Y_{19,21,23}$	--	--
	Cigarette use	B_{19-23}	$Y_{19,21,23}$	$B_{17,20-23}$	$Y_{17,21,23}$	--	--	B_{19-23}	$Y_{19,21,23}$	B_{19-23}	$Y_{19,21,23}$	--	--
Nebraska	E-cigarette use	B_{19-23}	$Y_{19,21,23}$	$B_{17,20-23}$	$Y_{17,21,23}$	--	--	$B_{19,20}$	Y_{19}	B_{20-23}	$Y_{21,23}$	--	--
	Cigarette use	B_{19-23}	$Y_{19,21,23}$	$B_{17,20-23}$	$Y_{17,21,23}$	--	--	$B_{19,20}$	Y_{19}	B_{20-23}	$Y_{21,23}$	--	--
Nevada	E-cigarette use	--	$Y_{19,21,23}$	$B_{17,20-23}$	$Y_{17,21,23}$	B_{20-23}	$Y_{21,23}$	B_{20}	Y_{19}	B_{20-23}	$Y_{21,23}$	--	--
	Cigarette use	B_{19-23}	$Y_{19,21,23}$	$B_{17,20-23}$	$Y_{17,21,23}$	B_{20-23}	$Y_{21,23}$	$B_{19,20}$	Y_{19}	B_{20-23}	$Y_{21,23}$	--	--
New Hampshire	E-cigarette use	B_{19-23}	$Y_{19,21,23}$	$B_{17,20-23}$	$Y_{17,21,23}$	B_{20-23}	$Y_{21,23}$	$B_{19,20}$	Y_{19}	B_{20-23}	$Y_{21,23}$	--	--
	Cigarette use	B_{19-23}	$Y_{19,23}$	$B_{17,20-23}$	$Y_{17,23}$	B_{20-23}	$Y_{21,23}$	$B_{19,20}$	Y_{19}	B_{20-23}	Y_{23}	--	--
New Mexico	E-cigarette use	--	$Y_{19,21,23}$	$B_{17,20-23}$	$Y_{17,21,23}$	B_{20-23}	$Y_{19,21,23}$	B_{20}	Y_{19}	B_{21-23}	$Y_{21,23}$	B_{20-23}	$Y_{19,21,23}$
	Cigarette use	B_{19-23}	$Y_{19,21,23}$	$B_{17,20-23}$	$Y_{17,21,23}$	B_{19-23}	$Y_{19,21,23}$	$B_{19,20}$	Y_{19}	B_{21-23}	$Y_{21,23}$	B_{19-23}	$Y_{19,21,23}$

© 2025 Cheng D et al. *JAMA Network Open*.

State	Outcome	Balanced[b]		Pre-year 2017[c]		EC taxation[d]		No T21[e]		Require Licensure[f]		EC clean indoor air[g]	
North Carolina	E-cigarette use	B_{19-23}	$Y_{19,21,23}$	$B_{17,20-23}$	$Y_{17,21,23}$	B_{19-23}	$Y_{19,21,23}$	B_{19-23}	$Y_{19,21,23}$	--	--	--	--
	Cigarette use	B_{19-23}	$Y_{19,21,23}$	$B_{17,20-23}$	$Y_{17,21,23}$	B_{19-23}	$Y_{19,21,23}$	B_{19-23}	$Y_{19,21,23}$	--	--	--	--
North Dakota	E-cigarette use	--	$Y_{19,21,23}$	$B_{17,20-23}$	$Y_{17,21,23}$	--	--	B_{20}	Y_{19}	--	--	B_{20-23}	$Y_{19,21,23}$
	Cigarette use	B_{19-23}	$Y_{19,21,23}$	$B_{17,20-23}$	$Y_{17,21,23}$	--	--	$B_{19,20}$	Y_{19}	--	--	B_{19-23}	$Y_{19,21,23}$
Ohio	E-cigarette use	--	$Y_{19,21}$	$B_{17,20-23}$	Y_{21}	B_{20-23}	$Y_{19,21}$	--	Y_{19}	--	--	$B_{22,23}$	--
	Cigarette use	B_{19-23}	$Y_{19,21}$	$B_{17,20-23}$	Y_{21}	B_{19-23}	$Y_{19,21}$	B_{19}	Y_{19}	--	--	$B_{22,23}$	--
Oklahoma	E-cigarette use	--	$Y_{19,21,23}$	$B_{17,21-23}$	$Y_{17,21,23}$	--	--	--	Y_{19}	--	--	--	--
	Cigarette use	B_{19-23}	$Y_{19,21,23}$	$B_{17,20-23}$	$Y_{17,21,23}$	--	--	B_{19}	Y_{19}	--	--	--	--
Oregon	E-cigarette use	B_{19-23}	--	$B_{17,20-23}$	--	B_{21-23}	--	--	--	$B_{22,23}$	--	B_{19-23}	--
	Cigarette use	B_{19-23}	--	$B_{17,20-23}$	--	B_{21-23}	--	--	--	$B_{22,23}$	--	B_{19-23}	--
Pennsylvania	E-cigarette use	--	$Y_{19,21,23}$	$B_{17,20-22}$	$Y_{17,21,23}$	B_{20-22}	$Y_{19,21,23}$	B_{20}	Y_{19}	B_{20-22}	$Y_{19,21,23}$	--	--
	Cigarette use	B_{19-22}	$Y_{19,21,23}$	$B_{17,20-22}$	$Y_{17,21,23}$	B_{19-22}	$Y_{19,21,23}$	$B_{19,20}$	Y_{19}	B_{19-22}	$Y_{19,21,23}$	--	--
South Carolina	E-cigarette use	--	$Y_{19,21}$	$B_{17,21-23}$	$Y_{17,21}$	--	--	B_{21-23}	$Y_{19,21}$	--	--	--	--
	Cigarette use	B_{19-23}	$Y_{19,21}$	$B_{17,20-23}$	$Y_{17,21}$	--	--	B_{19-23}	$Y_{19,21}$	--	--	--	--
South Dakota	E-cigarette use	B_{19-23}	$Y_{19,21}$	$B_{17,20-23}$	Y_{21}	--	--	$B_{19,20}$	Y_{19}	--	--	B_{20-23}	Y_{21}
	Cigarette use	B_{19-23}	$Y_{19,21}$	$B_{17,20-23}$	Y_{21}	--	--	$B_{19,20}$	Y_{19}	--	--	B_{20-23}	Y_{21}
Tennessee	E-cigarette use	B_{19-23}	$Y_{19,21,23}$	$B_{17,20-23}$	$Y_{17,21,23}$	--	--	$B_{19,20}$	Y_{19}	--	--	--	--
	Cigarette use	B_{19-23}	$Y_{19,21,23}$	$B_{17,20-23}$	$Y_{17,21,23}$	--	--	$B_{19,20}$	Y_{19}	--	--	--	--
Texas	E-cigarette use	B_{19-23}	$Y_{19,21,23}$	$B_{17,20-23}$	$Y_{17,21,23}$	--	--	B_{19-22}	$Y_{19,21}$	$B_{22,23}$	Y_{23}	--	--
	Cigarette use	B_{19-23}	$Y_{19,21,23}$	$B_{17,20-23}$	$Y_{17,21,23}$	--	--	B_{19-22}	$Y_{19,21}$	$B_{22,23}$	Y_{23}	--	--
Vermont	E-cigarette use	--	$Y_{19,21,23}$	$B_{17,20-23}$	$Y_{17,21,23}$	B_{20-23}	$Y_{19,21,23}$	--	Y_{19}	B_{20-23}	$Y_{19,21,23}$	B_{20-23}	$Y_{19,21,23}$
	Cigarette use	B_{19-23}	$Y_{19,21,23}$	$B_{17,20-23}$	$Y_{17,21,23}$	B_{19-23}	$Y_{19,21,23}$	B_{19}	Y_{19}	B_{19-23}	$Y_{19,21,23}$	B_{19-23}	$Y_{19,21,23}$
Virginia	E-cigarette use	B_{19-23}	$Y_{19,21,23}$	$B_{17,20-23}$	$Y_{17,21,23}$	B_{20-23}	$Y_{21,23}$	B_{19}	Y_{19}	--	--	--	--
	Cigarette use	B_{19-23}	$Y_{19,21,23}$	$B_{17,20-23}$	$Y_{17,21,23}$	B_{20-23}	$Y_{21,23}$	B_{19}	Y_{19}	--	--	--	--
Washington	E-cigarette use	--	--	$B_{17,20-23}$	--	B_{20-23}	--	--	--	B_{20-23}	--	--	--
	Cigarette use	B_{19-23}	--	$B_{17,20-23}$	--	B_{19-23}	--	B_{19}	--	B_{19-23}	--	--	--
West Virginia	E-cigarette use	--	$Y_{19,21}$	$B_{17,20-23}$	$Y_{17,21}$	B_{20-23}	$Y_{19,21}$	B_{20-23}	$Y_{19,21}$	--	--	--	--
	Cigarette use	B_{19-23}	$Y_{19,21}$	$B_{17,20-23}$	$Y_{17,21}$	B_{19-23}	$Y_{19,21}$	B_{19-23}	$Y_{19,21}$	--	--	--	--
Wisconsin	E-cigarette use	B_{19-23}	$Y_{19,21,23}$	$B_{17,20-23}$	$Y_{17,21,23}$	B_{19-23}	$Y_{19,21,23}$	B_{19-23}	$Y_{19,21,23}$	--	--	--	--
	Cigarette use	B_{19-23}	$Y_{19,21,23}$	$B_{17,20-23}$	$Y_{17,21,23}$	B_{19-23}	$Y_{19,21,23}$	B_{19-23}	$Y_{19,21,23}$	--	--	--	--
Wyoming	E-cigarette use	B_{19-23}	--	$B_{17,20-23}$	--	B_{20-23}	--	$B_{19,20}$	--	--	--	--	--
	Cigarette use	B_{19-23}	--	$B_{17,20-23}$	--	B_{20-23}	--	$B_{19,20}$	--	--	--	--	--

[a] Columns for each sensitivity analysis type indicate which state-year combination contributed EC and/or cigarette use data. Subscripts indicate which years of data are included for each survey type: "Y_{xx}" for YRBS and "B_{xx}" for BRFSS. For example, a state might contribute EC YRBS use data in 2019 and 2021 to a particular sensitivity analysis, which would be captured by the notation: "$Y_{19,21}$". EC=E-cigarette

[b] **Balanced panel sensitivity analysis:** Available state EC and/or cigarette use data were included in the balanced panel sensitivity analysis only if data were available for both the pre-policy year of 2019 and for each post-policy year (2020-2023).

[c] **Pre-year 2017 sensitivity analysis:** Available state EC and/or cigarette use data were included in the pre-2017 sensitivity analysis, which used 2017 instead of 2019 as the pre-policy year.

© 2025 Cheng D et al. *JAMA Network Open*.

[d] **EC taxation sensitivity analysis**: Available state EC and/or cigarette use data were included in the EC taxation sensitivity analysis only for states in years with EC taxes present.
[e] **No T21 sensitivity analysis**: Available state EC and/or cigarette use data were included in the T21 sensitivity analysis only for states in years without a "tobacco 21" law restricting sales of ECs and cigarettes for individuals 18-20.
[f] **Require Licensure sensitivity analysis:** Available state EC and/or cigarette use data were included in the licensure sensitivity analysis only for states in years requiring retail licensure to sell ECs.
[g] **EC clean indoor air sensitivity analysis**: Available state EC and/or cigarette use data were included in the EC clean indoor air sensitivity analysis only for states in years with prohibitions on EC use at restaurants, workplaces, and bars.

© 2025 Cheng D et al. *JAMA Network Open*.

eTable 8: Sensitivity analyses for association of flavor restriction policy with current e-cigarette (EC) and cigarette use by age group (numerical estimates)

		E-cigarette use		Cigarette use	
	Year	ATT (95% CI)	p-value	ATT (95% CI)	p-value
Youth (Grade 9-12, YRBS)					
Balanced	2021	-0.018 (-0.072 to 0.037)	0.46	0.017 (0.006 to 0.028)	0.008
Pre-year 2017		-0.022 (-0.068 to 0.024)	0.29	0.025 (0.006 to 0.043)	0.02
EC taxation		-0.016 (-0.126 to 0.094)	0.62	0.024 (-0.004 to 0.053)	0.07
No T21		-0.005 (-0.179 to 0.169)	0.91	0.013 (-0.014 to 0.039)	0.18
Require licensure		0.023 (-0.114 to 0.159)	0.68	0.022 (0.002 to 0.042)	0.04
EC clean indoor air		0.02 (-0.065 to 0.104)	0.61	0.025 (0.004 to 0.046)	0.02
Balanced	2023	-0.008 (-0.056 to 0.04)	0.69	0.008 (-0.002 to 0.017)	0.1
Pre-year 2017		-0.029 (-0.075 to 0.016)	0.17	0.013 (0 to 0.027)	0.06
EC taxation		-0.011 (-0.135 to 0.113)	0.77	0.015 (-0.023 to 0.053)	0.26
Require licensure		0.018 (-0.11 to 0.145)	0.74	0.01 (-0.009 to 0.029)	0.24
EC clean indoor air		0.016 (-0.063 to 0.095)	0.65	0.014 (-0.011 to 0.038)	0.23
Young adult (Age 18-24, BRFSS)					
Balanced	2020	-0.043 (-0.098 to 0.012)	0.1	0.016 (0.002 to 0.03)	0.03
Pre-year 2017		-0.023 (-0.053 to 0.006)	0.1	0.009 (-0.014 to 0.033)	0.37
EC taxation		-0.079 (-0.144 to -0.013)	0.02	0.001 (-0.026 to 0.028)	0.93
No T21		-0.019 (-0.083 to 0.045)	0.45	0.02 (0.001 to 0.04)	0.05
Require licensure		-0.027 (-0.09 to 0.036)	0.33	0.017 (-0.003 to 0.037)	0.08
EC clean indoor air		-0.081 (-0.174 to 0.011)	0.08	0.01 (-0.026 to 0.045)	0.54
Balanced	2021	-0.037 (-0.107 to 0.034)	0.25	0.04 (0.025 to 0.055)	<0.001
Pre-year 2017		-0.027 (-0.06 to 0.006)	0.09	0.033 (0.009 to 0.056)	0.01
EC taxation		-0.093 (-0.15 to -0.036)	0.004	0.019 (-0.007 to 0.045)	0.13
No T21		-0.015 (-0.197 to 0.167)	0.77	0.052 (0.01 to 0.094)	0.03
Require licensure		-0.019 (-0.099 to 0.061)	0.57	0.038 (0.017 to 0.059)	0.007
EC clean indoor air		-0.097 (-0.194 to 0)	0.05	0.045 (0.011 to 0.079)	0.02
Balanced	2022	-0.049 (-0.103 to 0.004)	0.07	0.028 (0.013 to 0.042)	0.004
Pre-year 2017		-0.05 (-0.078 to -0.022)	0.004	0.023 (-0.002 to 0.048)	0.07
EC taxation		-0.113 (-0.174 to -0.051)	0.002	0.01 (-0.015 to 0.035)	0.37
Require licensure		-0.045 (-0.115 to 0.026)	0.17	0.031 (0.001 to 0.06)	0.05
EC clean indoor air		-0.119 (-0.221 to -0.018)	0.03	0.033 (0.001 to 0.065)	0.05
Balanced	2023	-0.042 (-0.107 to 0.024)	0.17	0.028 (0.011 to 0.046)	0.009
Pre-year 2017		-0.043 (-0.078 to -0.009)	0.02	0.028 (0 to 0.056)	0.05
EC taxation		-0.102 (-0.166 to -0.038)	0.005	0.022 (-0.006 to 0.049)	0.11
Require licensure		-0.047 (-0.135 to 0.041)	0.24	0.034 (-0.001 to 0.068)	0.05
EC clean indoor air		-0.119 (-0.221 to -0.018)	0.03	0.033 (0.001 to 0.065)	0.05

© 2025 Cheng D et al. *JAMA Network Open*.

		E-cigarette use		Cigarette use	
	Year	ATT (95% CI)	p-value	ATT (95% CI)	p-value
		Adult (Age ≥25, BRFSS)			
Balanced	2020	0 (-0.012 to 0.011)	0.93	0.001 (-0.011 to 0.012)	0.89
Pre-year 2017		0.002 (-0.006 to 0.01)	0.53	-0.004 (-0.014 to 0.007)	0.45
EC taxation		0.007 (-0.008 to 0.023)	0.3	-0.015 (-0.04 to 0.01)	0.19
No T21		0.001 (-0.023 to 0.025)	0.9	-0.006 (-0.032 to 0.021)	0.56
Require licensure		-0.008 (-0.021 to 0.005)	0.18	0.003 (-0.031 to 0.038)	0.81
EC clean indoor air		-0.003 (-0.019 to 0.012)	0.63	0 (-0.017 to 0.016)	0.96
Balanced	2021	-0.006 (-0.019 to 0.006)	0.26	0.001 (-0.01 to 0.013)	0.75
Pre-year 2017		-0.006 (-0.014 to 0.003)	0.15	-0.002 (-0.011 to 0.008)	0.72
EC taxation		-0.001 (-0.015 to 0.012)	0.83	-0.009 (-0.032 to 0.013)	0.38
No T21		-0.016 (-0.041 to 0.01)	0.13	0.004 (-0.057 to 0.065)	0.82
Require licensure		-0.015 (-0.028 to -0.001)	0.04	0.003 (-0.033 to 0.038)	0.86
EC clean indoor air		-0.01 (-0.027 to 0.007)	0.2	0.002 (-0.013 to 0.017)	0.73
Balanced	2022	-0.006 (-0.021 to 0.009)	0.35	0.005 (-0.005 to 0.016)	0.24
Pre-year 2017		-0.007 (-0.016 to 0.002)	0.13	0.002 (-0.006 to 0.011)	0.5
EC taxation		-0.001 (-0.015 to 0.013)	0.83	-0.008 (-0.032 to 0.015)	0.44
Require licensure		-0.016 (-0.029 to -0.003)	0.02	0.009 (-0.026 to 0.045)	0.52
EC clean indoor air		-0.008 (-0.028 to 0.011)	0.33	-0.002 (-0.019 to 0.015)	0.8
Balanced	2023	-0.008 (-0.016 to -0.001)	0.04	0.007 (-0.005 to 0.019)	0.19
Pre-year 2017		-0.009 (-0.015 to -0.003)	0.01	0.002 (-0.009 to 0.014)	0.64
EC taxation		-0.003 (-0.015 to 0.008)	0.5	-0.008 (-0.029 to 0.014)	0.45
Require licensure		-0.019 (-0.031 to -0.007)	0.009	0.007 (-0.032 to 0.047)	0.64
EC clean indoor air		-0.008 (-0.028 to 0.011)	0.33	-0.002 (-0.019 to 0.015)	0.8

© 2025 Cheng D et al. *JAMA Network Open*.

eFigure 1: State-level trends in current e-cigarette and cigarette use over time by age group

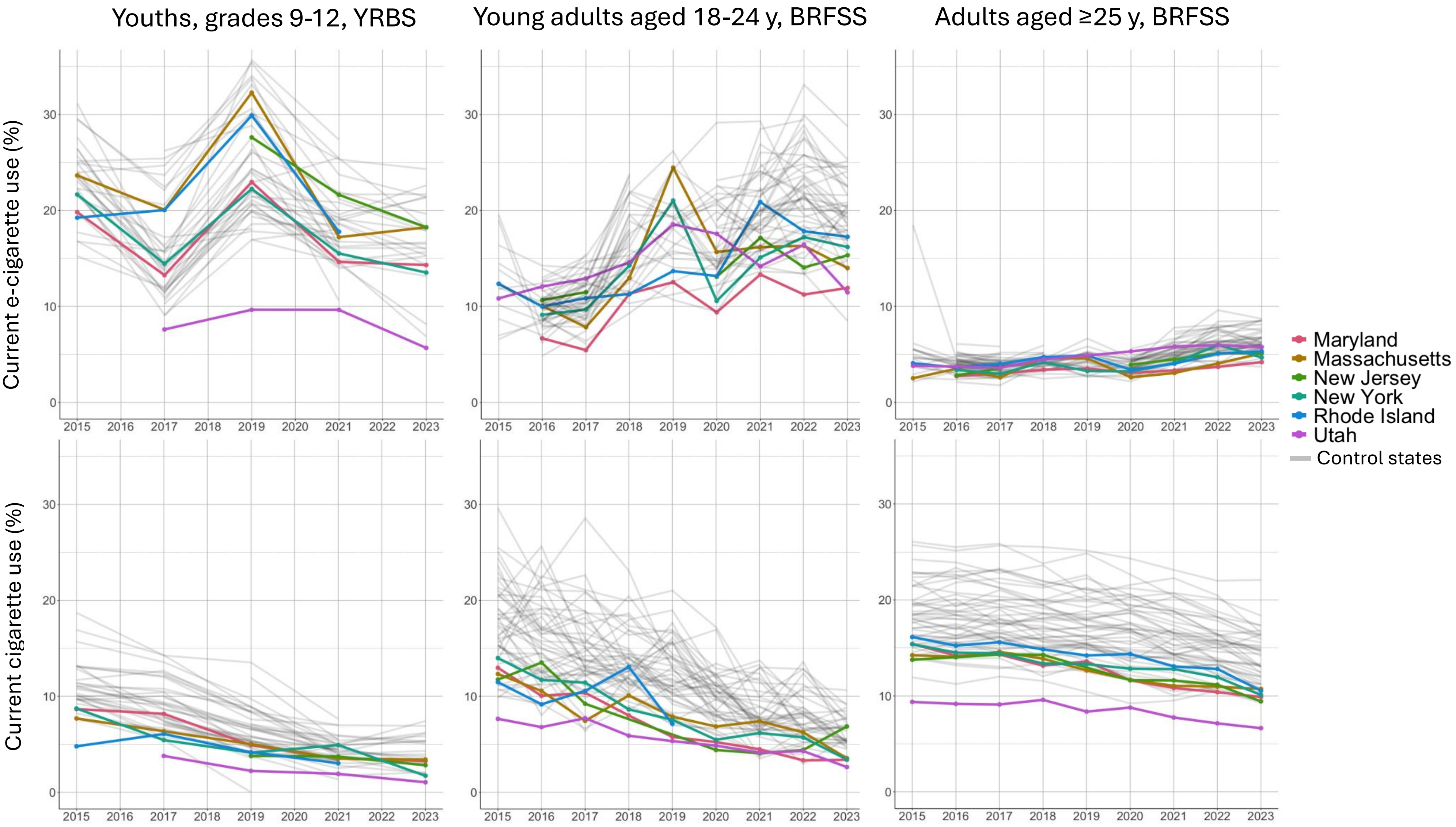

Current e-cigarette and cigarette use prevalence estimated based on self-reported past 30 day use in youth and use on some or every day in young adults and adults. Respondents with missing, refused, or "don't know" responses are excluded.

Prevalence are calculated using complex survey weights to obtain state-representative estimates in each year.

© 2025 Cheng D et al. *JAMA Network Open*.

Prevalence estimates in state-years based on < 50 respondents and relative standard error (standard error/estimate; RSE) >30% among young adults and adults are excluded per CDC BRFSS data suppression rules.

Prevalence estimates of current e-cigarette use for DE in 2015 and 2019 are suppressed as the RSE >10% among young adults and adults. The prevalence estimate of current e-cigarette use for RI in 2015 among young adults may be questionable as it had a RSE between 20-30%. The prevalence estimate of current e-cigarette use for CT in 2016 needs to be interpreted with caution as its RSE was between 15-30%.

© 2025 Cheng D et al. *JAMA Network Open*.

eFigure 2: Event-study plots for e-cigarette and cigarette ATT estimates by age group

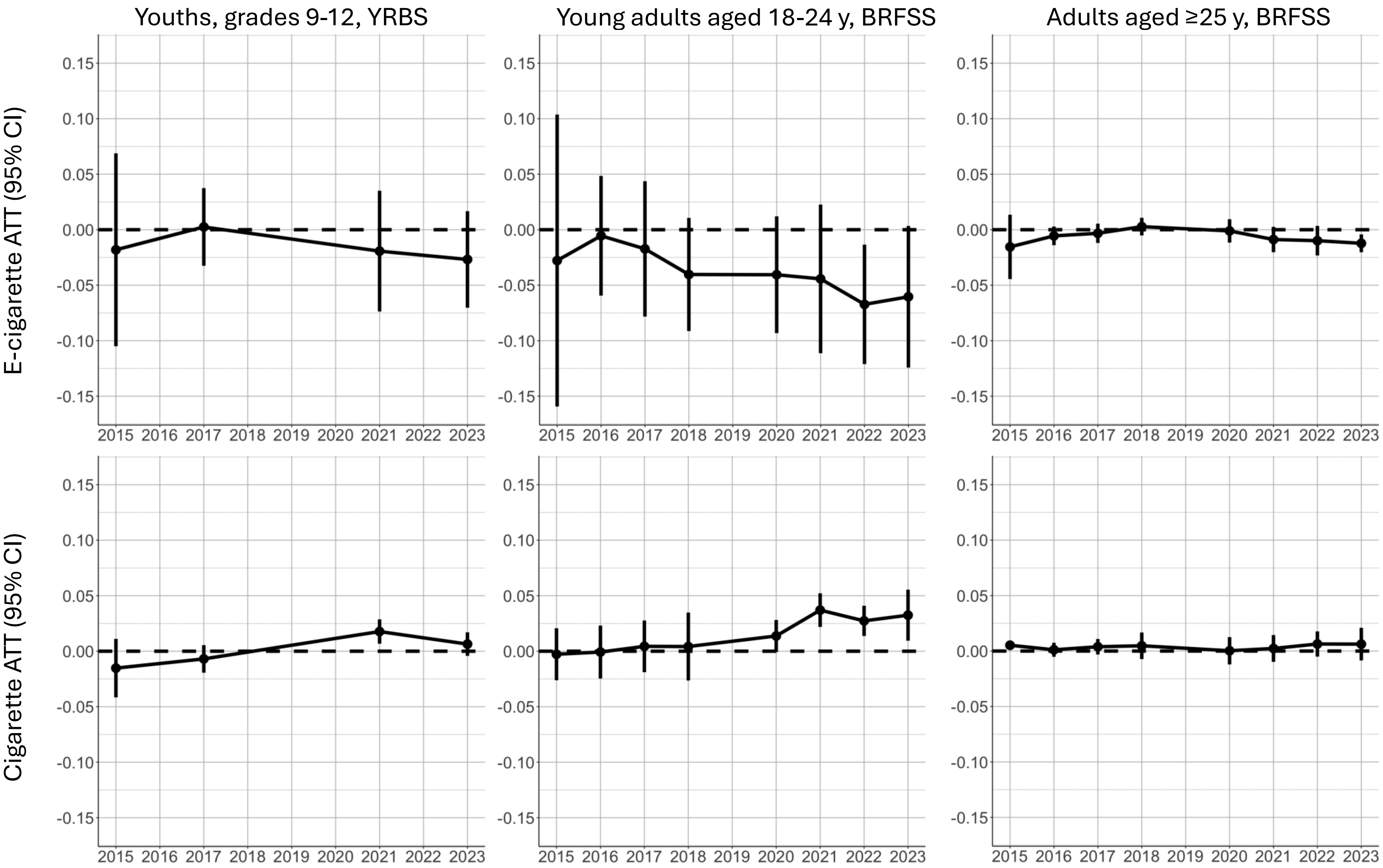

ATT=average treatment effect among treated.

© 2025 Cheng D et al. *JAMA Network Open*.
