# Supplementary material for: State E-Cigarette Flavor Restrictions and Tobacco Product Use in Youths and Adults
Source: JAMA Netw Open. 2025 Jul 30;8(7):e2524184. doi: 10.1001/jamanetworkopen.2025.24184 (PMC12311715; doi:10.1001/jamanetworkopen.2025.24184)
Supplement: Supplement 2. — Data Sharing Statement [file jamanetwopen-e2524184-s002.pdf]

Data Sharing Statement

Cheng. State E-Cigarette Flavor Restrictions and Tobacco Product Use in Youths and Adults. JAMA Netw Open. Published online July 30, 2025. doi:10.1001/jamanetworkopen.2025.24184

Data
Data available: No

Additional Information
Explanation for why data not available: BRFSS and YRBS data and data dictionaries can be accessed through the CDC's website: www.cdc.gov. Data for additional questions administered by states themselves can be requested by contacting each state's BRFSS and/or YRBS coordinator.
